# Synthesis and Characterization of Novel Uracil-Modified Chitosan as a Promising Adsorbent for Efficient Removal of Congo Red Dye

**DOI:** 10.3390/polym14020271

**Published:** 2022-01-10

**Authors:** Nouf F. Al-Harby, Ebtehal F. Albahly, Nadia A. Mohamed

**Affiliations:** 1 Department of Chemistry, College of Science, Qassim University, P.O. Box 6644, Buraydah 51452, Qassim, Saudi Arabia; 381225524@qu.edu.sa (E.F.A.); NA.AHMED@qu.edu.sa (N.A.M.); 2 Department of Chemistry, Faculty of Science, Cairo University, Giza 12613, Egypt

**Keywords:** uracil-modified chitosan adsorbent, Congo red dye, adsorption kinetics, adsorption isotherms, adsorption thermodynamic

## Abstract

Novel Uracil-modified chitosan (UCs) adsorbent has successfully been synthesized through a four-step method during which the amino groups of chitosan have been protected, then epoxy nuclei have been incorporated, afterwards the latter have been opened using 6-amino-1,3-dimethyl uracil, and finally the amino groups have been regained via removing the protection. Its structure was checked using FTIR, XRD and SEM techniques. The adsorption capacity of UCs for anionic Congo Red (CR) dye was studied under various conditions. It decreased significantly with increasing the solution pH value and dye concentration, while increased with increasing temperature. The adsorption of UCs for CR dye at different temperatures, solution pH and dye concentrations fitted to the kinetic model of pseudo-second order and Elovich model. The intraparticle diffusion model showed that the adsorption process involves multi-step process. The isotherm of CR dye adsorption by UCs conforms to the Langmuir isotherm model indicating the monolayer nature of adsorption. The maximum monolayer coverage capacity, q_max_, was 434.78 mg g^−1^. Studying the thermodynamic showed that the adsorption of CR dye onto UCs was endothermic as illustrated from the positive value of enthalpy (21.37 kJ mol^−1^). According to the values of *ΔG°*, the adsorption process was spontaneous at all selected temperatures. The value of *ΔS°* showed an increase in randomness for the adsorption of CR dye by UCs. The value of activation energy was 18.40 kJ mol^−1^.

## 1. Introduction

Pollution is considered one of the most serious environmental problems; as population growth is increasing over time, the pollution problem is also increasing [1]. Water can be polluted by different ways such as municipal and industrial discharge, spills, runoff and deposition of various pollutants [2]. In between various organic pollutants found the synthetic dyes which are used in various applications such as printing of papers, textile, leathers, photography, nutriment, cosmetics as well as medicinal drugs [3]. A very little amount of a dye in water has a high discernible and inadmissible effect. Various studies reported the toxicity of these dyes, which can cause severe harmful effects on human health such as heart defects, cancer, skin irritation and mutations [1,3]. Additionally, the reactive dyes can affect the immune system in severe cases that is due to the reaction of human body dramatically with dye molecules after their inhalation [3]. On the other hand, one of the most highly toxic dyes are the azo dyes that are released in high amounts into water system. The carcinogenic effect of the azo dyes was reported as they are associated with splenic sacromas, bladder cancer and hepatocarcinomas. Due to that these dyes are made of carcinogens such as benzidine which accumulates causing a serious threat effect onto human body [1,2,3].

Among different azo dyes found the Congo red dye (CR) [1-naphthalene sulfonic acid, 3,3′(4,4′biphenylenebis(azo))bis(4-amino-)disodium salt]. It is a benzidine-based anionic diazo dye. It can be prepared by a reaction of tetrazotized benzidine with naphthionic acid. Although it is highly carcinogenic and has been banned in various countries, it is still widely used in many countries till now. It has a very stable structure; this stability makes it high toxic as it has high resistance against degradation. It is the most commonly used dye in textile applications, it can be metabolized into benzidine which is known with its mutagenity and carcinogenity.

There are many methods were used for water treatment such as coagulation/flocculation, electro-dialysis, reverse osmosis, oxidation, ion exchange, ozone treatment and photocatalytic degradation [4,5,6]. Nowadays, adsorption considered a competitive method for removal of contaminants from water [7,8]. There are numerous types of adsorbents such as alumina, clays, carbon nanotubes, graphene, and zeolite. Recently, there is a high need for low-cost and non-conventional adsorbents, especially the natural materials that act as biosorbents [9].

Chitin is the second abundant natural polysaccharide biopolymer after cellulose. It is found in the exoskeleton of cartilages of mollusks, crustaceans, insects and cell walls of micro-organisms. Chitosan is deacetylated form of the natural biopolymer chitin. It consists of 2-acetamido-2-deoxy-β-D-glucopyranose and 2-amino-2-deoxy-β-D-glucopyranose linked by β(1,4)-linkage [10]. Chitosan possesses important properties more than chitin. It has common properties similar to biopolymers including non-toxicity, biodegradability and biocompatibility [10]. Chitosan possesses positive charges on its surface that helps it to bind with negative charge species. In this respect, it can be strongly used in water remediation applications [10]. Although chitosan possesses so many advantages and is high potential, it faces many drawbacks such as high solubility and low stability in acidic medium, low thermal resistance and low porosity. Consequently, this causes high impediment for a lot of applications, particularly when it is used for adsorbing dyes from their aqueous solutions due to the dye effluents is usually acidic. To overcome these problems, it is important to apply chemical modifications on chitosan chains. Grafting copolymerization [11], blending with other polymer [12] and crosslinking [13,14] techniques are applied for improving the properties of chitosan such as introducing an additional functional group, reducing its solubility and increasing its stability in acidic solutions, restraining its rate of degradation and boosting the life span of its products in diversified media. Although, there is a vast number of scientific publications studying chitosan modification to produce various number of chitosan derivatives, there is no studies reported the use of chitosan derivatives modified by uracil moiety as an adsorbent for water remediation.

In the present work, for averting the aforementioned shortcomings of chitosan and enhancing its capacity for adsorbing the anionic dyes from their aqueous solutions for industrial wastewater treatment, uracil moieties have been incorporated as pendant groups on the chitosan chains. This was achieved by four-step reaction; by protecting the amino groups of chitosan by their conversion into Schiff’s base, followed by incorporating epoxy moieties via reaction of epichlorohydrin with the hydroxyl groups on C-6 of chitosan Schiff’s base, then the epoxide moieties are easily opened using 6-amino-1,3-dimethyl uracil through a free electron pair of its amino group, and finally by removing the protection of the amnio groups and restoring them to get uracil-modified chitosan. The incorporation of nitrogen-rich uracil nuclei as pendant groups on the repeating units of chitosan, in addition to the restored amino groups, would increase the number of the basic centers available to adsorb the anionic dyes as Congo red dye. The effect of various variables on adsorption process have been investigated like temperature, initial dye concentration, contact time and pH of the adsorption medium for determining the best adsorption conditions and reaching to maximum adsorption capacity. The entropy, enthalpy and free energy of the adsorption were also estimated. This work was extended to study the possibility to regenerate the prepared adsorbent for its reusing.

## 2. Materials and Methods

### 2.1. Materials

**Figure 1:**
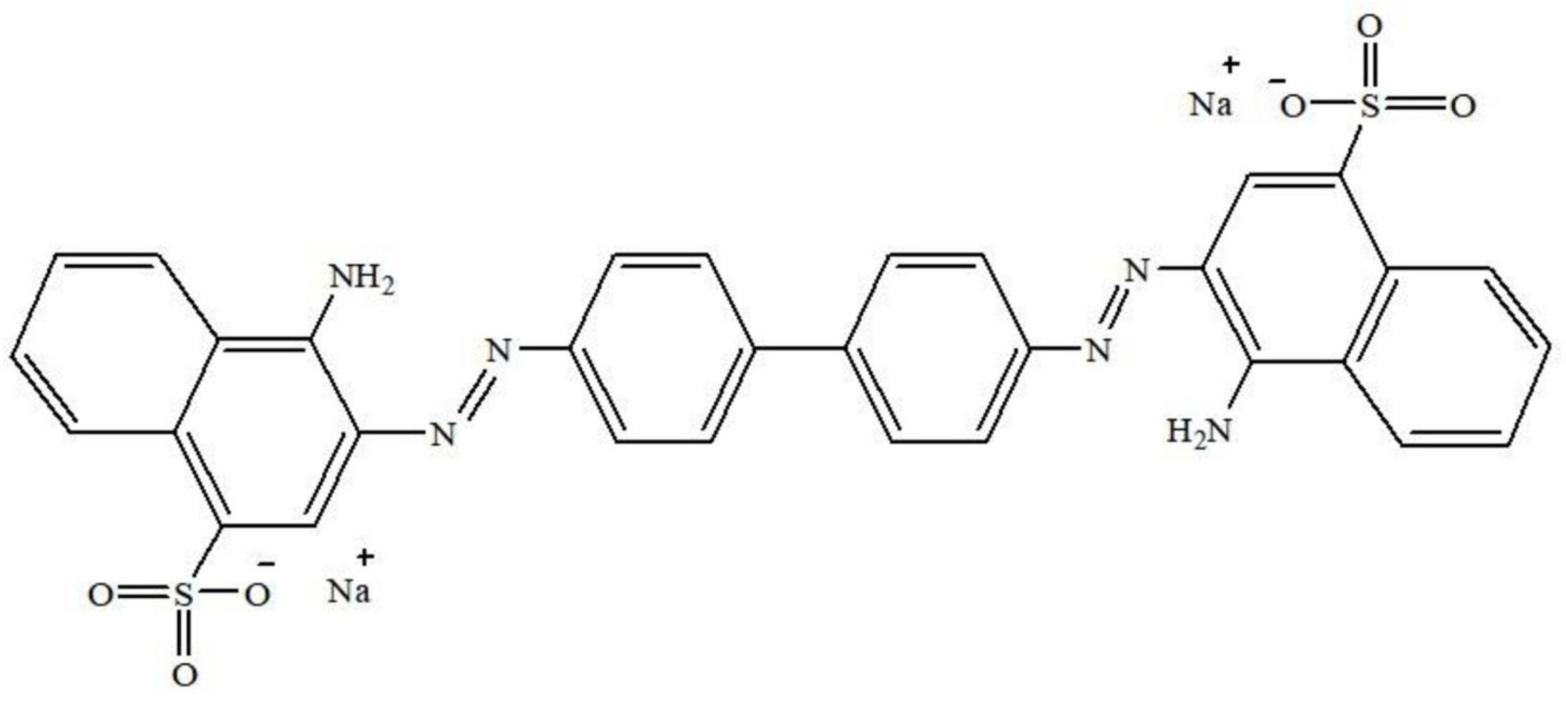
Chemical structure of CR dye.

Chitosan (1.0–3.0 × 10^5^ g mol ^−1^ and 98% deacetylation degree) was obtained from (Morris Plains, NJ, USA). Benzaldehyde and epichlorohydrin were purchased from PanReac. AppliChem-ITW Reagent (Darmstadt, Germany). 6-Amino-1,3-dimethyl uracil was supplied by Sigma-Aldrich (Munich, Germany). Congo red dye (CR) dye (Figure 1) was supplied by Winlab (Leicestershire, UK). The other chemicals and solvents were obtained from Sigma-Aldrich (Munich, Germany).

### 2.2. Preparation of Novel Uracil-Modified Chitosan (UCs) Adsorbent

First step: chitosan Schiff’s base should be prepared via reaction of chitosan with benzaldehyde to direct the chemical modification to the primary-OH groups on chitosan. The suspended chitosan (5 g) that swollen in 50 mL methanol was well stirred with 20 mL of benzaldehyde at room temperature for 24 h. The produced chitosan Schiff’s base was filtered, rinsed repeatedly with MeOH and dried at 50 °C to constant weight [15].

Second step: 4 g of chitosan Schiff’s base were suspended and well stirred in aqueous sodium hydroxide solution (120 mL, 0.001 mol L^−1^) at room temperature for 15 min for swelling. To the suspended solution, 10 mL of epichlorohydrin were slowly added with continuous stirring for further 6 h. The yielded epoxy chitosan Schiff’s base was filtered, rinsed repeatedly with H_2_O then methanol and dried at 50 °C to constant weight [16].

Third step: 2 g of 6-Amino-1,3-dimethyl uracil was dissolved in 25 mL of water, then was slowly mixed with a solution of epoxy chitosan Schiff’s base (2 g) that swelled and suspended in 60 mL of aqueous sodium hydroxide solution (0.001 mol L^−1^) and stirred at room temperature overnight. The produced uracil chitosan Schiff’s base was filtered, washed frequently with H_2_O then methanol and dried at 50 °C to constant weight.

**Scheme 1:**
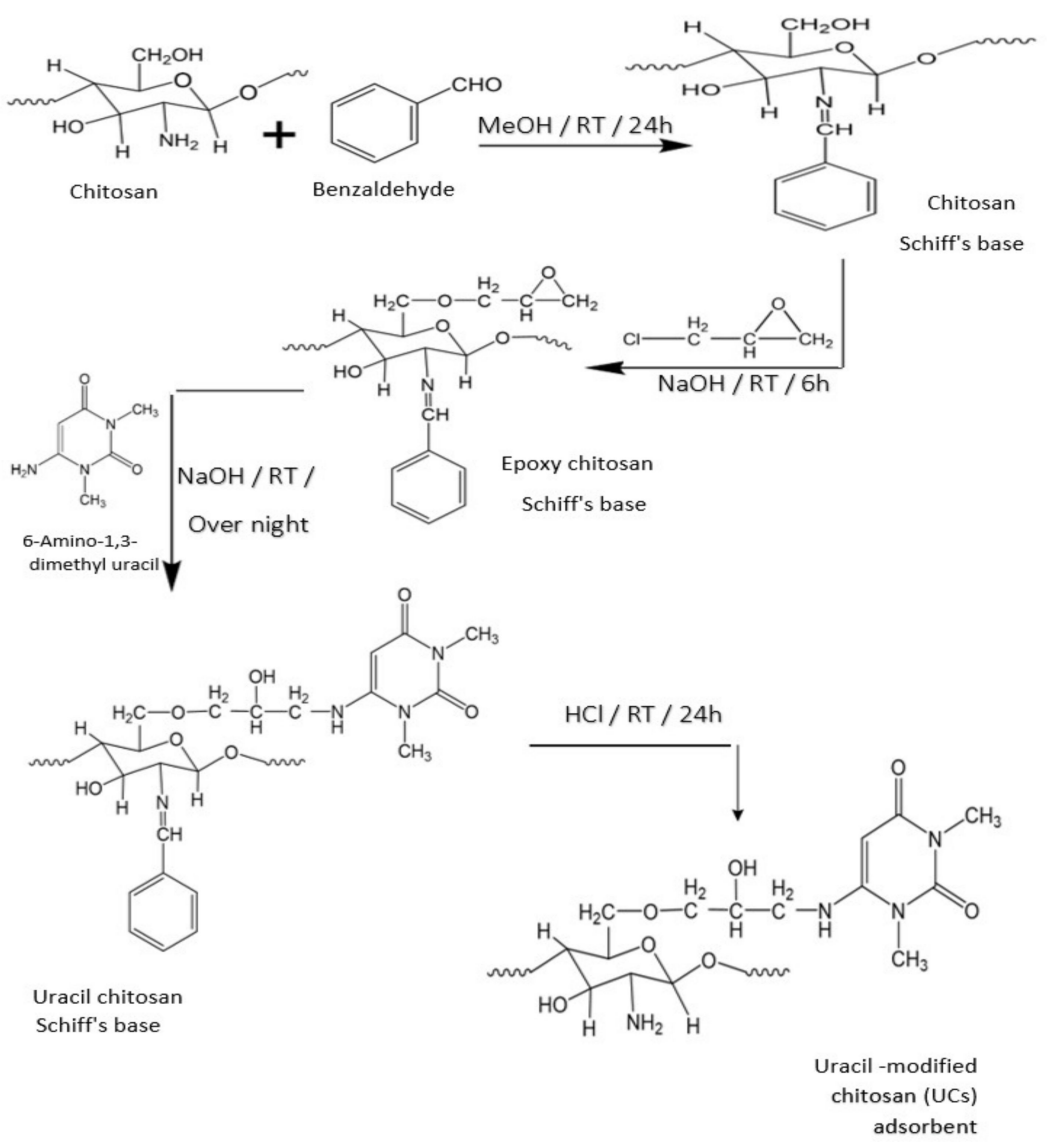
Preparation of novel UCs adsorbent.

Forth step: to remove the protection from the NH_2_ groups, uracil chitosan Schiff’s base (2 g) was treated with 60 mL of hydrochloric acid in ethanol (0.24 mol L^−1^ HCl) and stirring at room temperature for 24 h. The formed uracil-modified chitosan (UCs) adsorbent was neutralized with aqueous sodium carbonate solution (1 wt%) till pH 7, filtered, washed frequently with H_2_O then methanol and dried at 50 °C to constant weight (Scheme 1).

### 2.3. Measurements

#### 2.3.1. FTIR Spectroscopy

A Thermo Scientific Nicolet 6700 FTIR spectrometer (Yokohama, Japan) was utilized to record FTIR spectra of the modified chitosan derivatives using KBr pellets in the wave number range of 4000–500 cm^−1^.

#### 2.3.2. X-ray Diffractometry

A Rigaku Ultima-IV wide-angle X-ray diffractometer (Tokyo, Japan) was used to study the morphology of the modified chitosan derivatives at diffraction angles (2θ) over a range between 5 and 80° with a speed of 5° min^−1^.

#### 2.3.3. Scanning Electron Microscopy

Field emission scanning electron microscope JSM-7610F (Jeol, Freising, Germany) was used to photograph the surface topography of the modified chitosan derivatives after coating with a thin layer of gold at an accelerating voltage of 15 kV and at a magnification of 8000×.

### 2.4. Adsorption Studies

#### 2.4.1. Standard Curve of CR Dye

Anionic CR dye was chosen due to its extensive applications. Its stock solution was prepared by dissolution of CR dye in double distilled water of a concentration of 1000 mg L^−1^. The prepared stock solution was then diluted into 10 different dye concentrations using double distilled water. The absorbance of each dye solution using UV–vis spectrophotometer (Shimadzu UV/Vis 1601 spectrophotometer, Japan) at λ_max_ = 497 nm was measured. A calibration curve was plotted between concentration and absorbance using the predetermined concentrations of CR dye. The molar absorptivity was determined using Beer-Lambert law Equation (1).

A = εlc(1)

where A is the absorbance, ε is the molar absorptivity (L mol^−1^ cm^−1^), l is the path length of the cuvette that containing the sample (cm) and c is the concentration of dye in solution (mg L^−1^). The molar absorbance coefficient of CR dye was 0.045 L mol^−1^ cm^−1^.

#### 2.4.2. Adsorption of CR Dye Using UCs Adsorbent

The removal of CR dye by UCs adsorbent was studied using batch experiment. 50 mL of dye solution was added into an Erlenmeyer flask with 50 mg of the absorbent. The solution was then shaken in a water bath shaker (70 rpm) till attaining the equilibrium. The residual dye concentration was analyzed spectrophotometrically at λ_max_ = 497 nm.

The amount of adsorbed dye onto adsorbent at certain time, q_t_ (mg g^−1^), and at equilibrium, q_e_ (mg g^−1^), was calculated using Equations (2) and (3), respectively.

(2) $$q_{t}=\frac{\left(C_{o}-C_{t}\right)\;V}{W}$$

(3) $$q_{e}=\frac{\left(C_{o}-C_{e}\right)\;V}{W}$$

where q_t_ and q_e_ (mg g^−1^) are the amount of adsorbed dye at time t and at equilibrium, respectively, C_o_ (mg L^−1^) is the initial concentration of the dye, C_t_ (mg L^−1^) is the dye concentration at time t, V (L) is the volume of the dye solution and W (g) is the weight of the adsorbent.

The removal efficiency (R.E.) of dye can be calculated by Equation (4).

(4) $$\%\;R.E.=\frac{\left(C_{o}-C_{e}\right)}{C_{o}}\;\times \;100$$

where C_o_ is the initial concentration of the dye (mg L^−1^) and C_e_ is the equilibrium concentration of the dye (mg L^−1^).

Batch experiments were performed in stoppered Erlenmeyer flasks at various pH values (4, 7 and 9) using 50 mL of CR dye solution (600 mg L^−1^) and 50 mg of adsorbent. The solutions were continuously shaken at 70 rpm in a water bath shaker at 55 °C. For pH adjustment, HCl and NaOH solutions were utilized.

For studying the effect of temperature, 50 mg of UCs adsorbent was added to 50 mL of CR dye solution (600 mg L^−1^) at different temperatures (25, 35, 45 and 55 °C) at pH 9.

Studying the effect of CR dye concentration was carried out by adding 50 mg of UCs adsorbent to 50 mL of dye solution of various concentrations (400, 500, 600 and 1000 mg L^−1^) at pH 4 and 55 °C.

#### 2.4.3. Kinetic Studies

Studying kinetics of adsorption process is very significant for understanding the adsorption rate onto the particle surface, since adsorption kinetic shows the influence of different conditions on the speed of the process by using models that could describe this reaction. In addition, adsorption kinetic determines the mechanism of dye adsorption onto the adsorbent material.

The kinetic data of CR dye were modeled using four different kinetic models; pseudo-first order, Equation (5), pseudo-second order, Equation (6), Elovich, Equation (7), and intraparticle diffusion models, Equation (8).

(5) $$\log\left(q_{e}-q_{t}\right)=\log q_{e}-\frac{k_{1}}{2.303}t$$

where q_e_ and q_t_ (mg g^−1^) are the adsorption capacity at equilibrium and time t, respectively, k_1_ (min^−1^) is the pseudo first order rate constant and t is the time (min). The values of q_e_ and k_1_ can be determined from the intercept and the slope of the linear plot of log (q_e_ − q_t_) versus t.

(6) $$\frac{t}{q_{t}}=\frac{1}{k_{2}q_{e}^{2}}+\frac{t}{q_{e}}$$

where k_2_ (g mg^−1^ min^−1^) is the pseudo-second order constant [17].

(7) $$q_{t}=\frac{1}{\beta }\;\ln(\alpha \beta )+\frac{1}{\beta }\;\ln\;t$$

where α is the initial adsorption rate constant (mg g^−1^ min^−1^), β is the desorption constant related to the chemisorption activation energy and the surface coverage (g mg^−1^), and q_t_ (mg g^−1^) is the adsorbed dye at time t (min).

q_t_ = (k_int_ t^1/2^) + C (8)

where k_int_ (mg g^−1^ min^−1/2^) is the intraparticle diffusion constant, C (mg g^−1^) is a constant that is directly proportional to the boundary layer thickness [18].

To determine the most applicable model for describing the adsorption process, the normalized standard deviation (Δq_e_ (%) was calculated using Equation (9) [19].

(9) $$\Delta q_{e}\;(\%)=100\;\times \;\sqrt{\frac{{\left[\left(q_{t,\exp}-q_{t,\mathrm{cal}}\right)/q_{t,\exp}\right]}^{2}}{N-1}}$$

where q_t,exp_ (mg g^−1^) is the experimental adsorption capacity, q_t,cal_ (mg g^−1^) is the calculated adsorption capacity for pseudo-first and pseudo-second models and N is the number of data points.

#### 2.4.4. Adsorption Isotherm for CR Dye 9

Studying the adsorption isotherm is important for describing the ability of adsorbate molecules to distribute between the liquid and the solid phases at equilibrium state of the adsorption process. For studying adsorption isotherm, 10 mL of dye solution (400–1000 mg L^−1^) were shaken with 10 mg of adsorbent in a shaking water bath (70 rpm) at 55 °C and pH 9. After reaching the equilibrium, the concentrations of unadsorbed dye were determined by measuring absorbance of dye solution using UV-vis spectrophotometer at λ_max_ = 497 nm. The adsorption isotherm was studied using four models; Langmuir, Equation (10), Freundlich, Equation (12), Temkin, Equation (13), and Dubinin–Radushkevich models, Equation (14).

(10) $$\frac{C_{e}}{q_{e}}=\frac{1}{\left(q_{\max}K_{L}\right)}+\frac{C_{e}}{\left(q_{\max}\right)}$$

where C_e_ (mg L^−1^) is the equilibrium concentration of dye in solution, q_max_ (mg g^−1^) is the maximum monolayer coverage adsorption capacity, and K_L_ (L mg^−1^) is a Langmuir coefficient that is concerned with adsorption energy. There is an important parameter for Langmuir model which is the separation factor (R_L_) Equation (11).

(11) $$R_{L}=\frac{1}{\left(1+K_{L}C_{o}\right)}$$

This parameter indicates whether the process is irreversible, favorable, unfavorable and linear if R_L_ = 0, 0 < R_L_ < 1, R_L_ > 1 and R_L_ = 1, respectively.

(12) $$\ln q_{e}=\ln K_{F}+\frac{1}{n}\ln C_{e}$$

where K_F_ ((mg g^−1^) (L mg^−1^)^1/n^) is the adsorption capacity, while 1/n shows an indication of the favorability and feasibility of the adsorption process and the surface heterogeneity which is related to adsorption intensity.

q_e_ = B In K_T_ + B In (13)

where B (J mol^−1^) is the Temkin constant that is controlled by the adjusted uptake temperature, K_T_ (L g^−1^) is the binding constant of Temkin isotherm.

ln q_e_ = ln (X_m_) − βε^2^ (14)

where X_m_ (mg g^−1^) is monolayer saturation capacity, β (mol^2^ j^−2^) is the activity coefficient that is related to adsorption mean free energy and ε is the Polanyi potential which is determined by Equation (15).

(15) $$\varepsilon =\mathrm{RT}\;\ln\;(1+\frac{1}{C_{e}}\;)$$

where R (8.314 J mol^−1^K^−1^) is universal gas constant and T (K) is the absolute temperature.

The mean free energy E (kJ mol^−1^) of adsorption can be determined by Equation (16).

(16) $$E=\frac{1}{{\left(2\beta \right)}^{0.5}}$$

This mean free energy can determine the type of adsorption, if it is 8 < E < 16 kJ mol^−1^ the process is chemisorption, while if it is less than 8 kJ mol^−1^ the process is physisorption.

#### 2.4.5. Thermodynamic Studies

Thermodynamic parameters provide in-depth information of inherent energetic changes associated with adsorption; therefore, these parameters should be accurately evaluated. Studying thermodynamic parameters will give a better understanding of the adsorptive behavior for the dye towards adsorbents. Changes in Gibbs free energy, ∆G° (kJ mol^−1^), enthalpy change, ∆H° (kJ mol^−1^), and entropy change, ∆S° (J mol^−1^ K^−1^), were calculated for the adsorption of CR dye using four different temperatures (298, 308, 318 and 328 K) according to Equations (17) and (18).

∆G° = −RT ln K_c_ (17)

(18) $$\ln k_{c}=\frac{\Delta s{}^{\circ}}{R}-\frac{\Delta H{}^{\circ}}{\mathrm{RT}}$$

where R (8.314 J mol^−1^ K^−1^) is the universal gas constant, T (K) is the absolute temperature and K_c_ (q_e_/C_e_) is the distribution coefficient. By plotting ln K_c_ versus 1/T, the thermodynamic parameters can be tabulated.

#### 2.4.6. Activation Energy

Studying the activation energy (E_a_) was carried out by Arrhenius equation, which refers to the minimum energy for proceeding the reaction as shown in Equation (19).

(19) $$\ln\;k=\ln\;A-\frac{E_{a}}{\mathrm{RT}}$$

where A is the Arrhenius factor, R is the universal gas constant (8.314 J mol^−1^ K^−1^), E_a_ is the activation energy (kJ mol^−1^) and T is the absolute temperature (K). By plotting ln k (the pseudo-second order constant) versus inverse temperature (1/T), a straight line is obtained with slope–E_a_/R where E_a_ can be determined.

#### 2.4.7. Desorption Study

The dye desorption from adsorbent was performed by its washing with distilled water to remove any un-adsorbed dye molecules. Afterwards, the adsorbent (0.01 g) was immersed in 10 mL of desorption medium (ethanol, methanol, acetone or 0.1 N aqueous NaOH solution) at 25 °C for 24 h. The amount of the desorbed dye can be calculated using Equation (20).

(20) $$\%\;\mathrm{Dye}\;\mathrm{desorption}\;=q_{d}/q_{a}\times 100$$

where q_d_ is the amount of desorbed dye from the adsorbent surface (mg g^−1^) and q_a_ is the amount of the dye adsorbed onto the adsorbent (mg g^−1^) [18].

## 3. Results and Discussion

### 3.1. Synthesis of Novel UCs Adsorbent

The UCs adsorbent (Scheme 1) was prepared via a four-step procedure since the primary amine groups in chitosan were firstly protected by reacting with benzaldehyde for achieving chitosan Schiff’s base, in which the primary hydroxyl groups on C6 were reacted with epichlorohydrin for generating epoxy chitosan Schiff’s base, followed by reaction of the epoxy rings with 6-Amino-1,3-dimethyluracil for attaining uracil chitosan Schiff’s base, which is finally hydrolyzed in acidic medium to eliminate the benzaldehyde moieties and retrieve the amino groups to get UCs adsorbent. The amino and hydroxyl groups in addition to the basic functional groups of uracil incorporated into chitosan can potentially remove the acidic pollutants such as acidic dyes.

### 3.2. Characterization of Novel UCs Adsorbent

#### 3.2.1. FTIR Spectra of UCs Adsorbent

**Figure 2:**
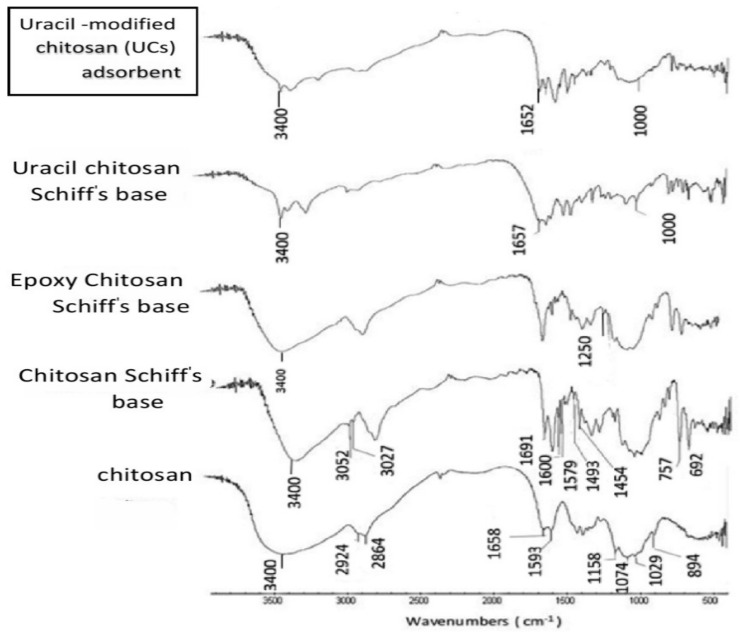
FTIR spectra of the modified chitosan derivatives.

FTIR spectra of chitosan and its modified derivatives were demonstrated in Figure 2. In the spectrum of chitosan, the existence of the saccharide moieties has been confirmed by the appearance of four absorption peaks at 1158, 1074, 1029, and 894 cm^−1^. A dense broad absorption peak at around 3700 to 3000 cm^−1^ appeared, relating to the peak of –OH groups overlapped with that for –NH_2_ and their hydrogen bonds. The symmetric absorption peak corresponded to –CH and –CH_2_ groups in the pyranose rings appeared at 2924 and 2864 cm^−1^, respectively. The high extent of deacetylation of chitosan is confirmed by the appearance of two weak absorption peaks at 1658 and 1593 cm^−1^ assigning to amide I and amide II, respectively. The overlapping between the amino groups deforming vibration at 1600 cm^−1^ and the stretching vibration peak of amide I at 1658 cm^−1^ results in an intensive peak [14,20,21].

The spectrum of chitosan Schiff’s base displayed similar absorption peaks of chitosan in addition to some new peaks as follows: (1) at 3052 and 3027 cm^−1^ indicated to C–H groups in aromatic ring, (2) at 1691 cm^−1^ corresponded to C=N groups, (3) at 1600, 1579, 1493 and 1454 cm^−1^ related to C=C bond in aromatic rings, and (4) at 757 and 692 cm^−1^ (strong) due to mono-substituted benzene rings [13,17].

Epoxy chitosan Schiff’s base spectrum, in addition to the afore-mentioned peaks, displayed a new peak at 1250 cm^−1^ which is ascribed to the epoxide moieties [22]. The spectrum of the uracil chitosan Schiff’s base showed the disappearance of the peak corresponded to the epoxide linkages at 1250 cm^−1^ and the appearance of a new absorption peak at 1000 cm^−1^ assigned to the C–H stretching vibration of pyrimidine ring [23], The stretching vibration peak of C=O of the uracil moiety is overlapped with the absorption peak of the amide I of chitosan at 1657 cm^−1^.

Removal of benzaldehyde moieties to obtain UCs adsorbent is confirmed by the disappearance of the absorption bands of mono-substituted benzene ring at 757 and 692 cm^−1^.

#### 3.2.2. Powder X-ray Diffraction of UCs Adsorbent

**Figure 3:**
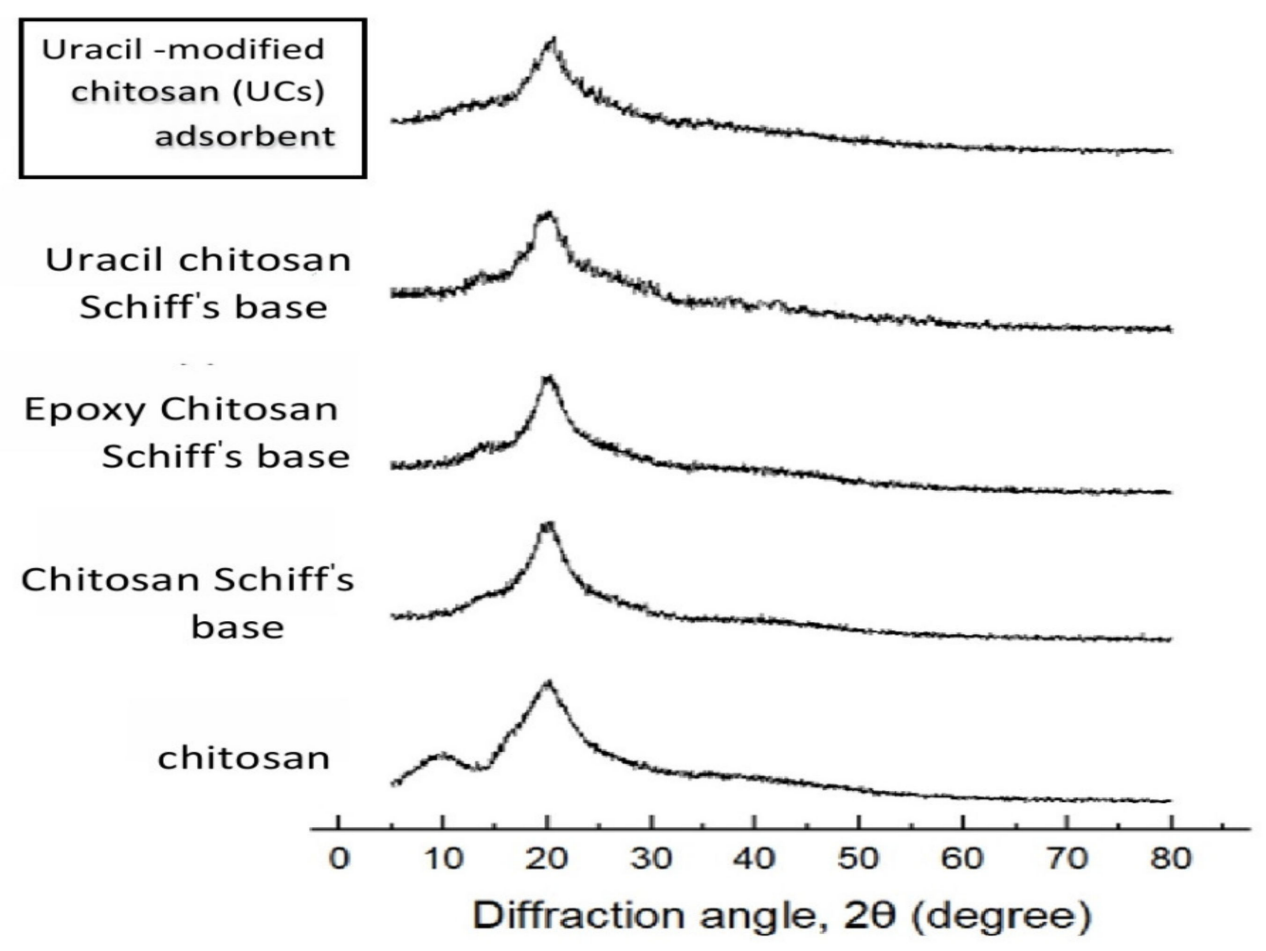
X-ray diffraction patterns of the modified chitosan derivatives.

X-ray diffractometry was used to explore the inner structures of the modified chitosan derivatives and their X-ray diffraction patterns were shown in Figure 3. There are two broad peaks in chitosan appeared near to 2θ = 10° and 20° which are attributable to its amorphous and crystalline regions, respectively [24]. This can be ascribed to the formation of the hydrogen bonds along its chains due to its possession of a great number of hydroxyl and amino groups. On the other hand, the modified chitosan derivatives are less crystalline than chitosan. This is illustrated by a disappearance of the peak at 2θ = 10° and a decrease of the intensity of the peak at 2θ = 20°. After modification of chitosan, its functionality greatly changed with a reduction of the hydrogen bonds due to consumption of its polar –NH_2_ and/or –OH groups and incorporation of the modifiers moieties that separate the chitosan chains away from each other. This leads to an increase of the amorphous region and a decrease of the crystalline region.

#### 3.2.3. SEM Analysis of UCs Adsorbent

**Figure 4:**
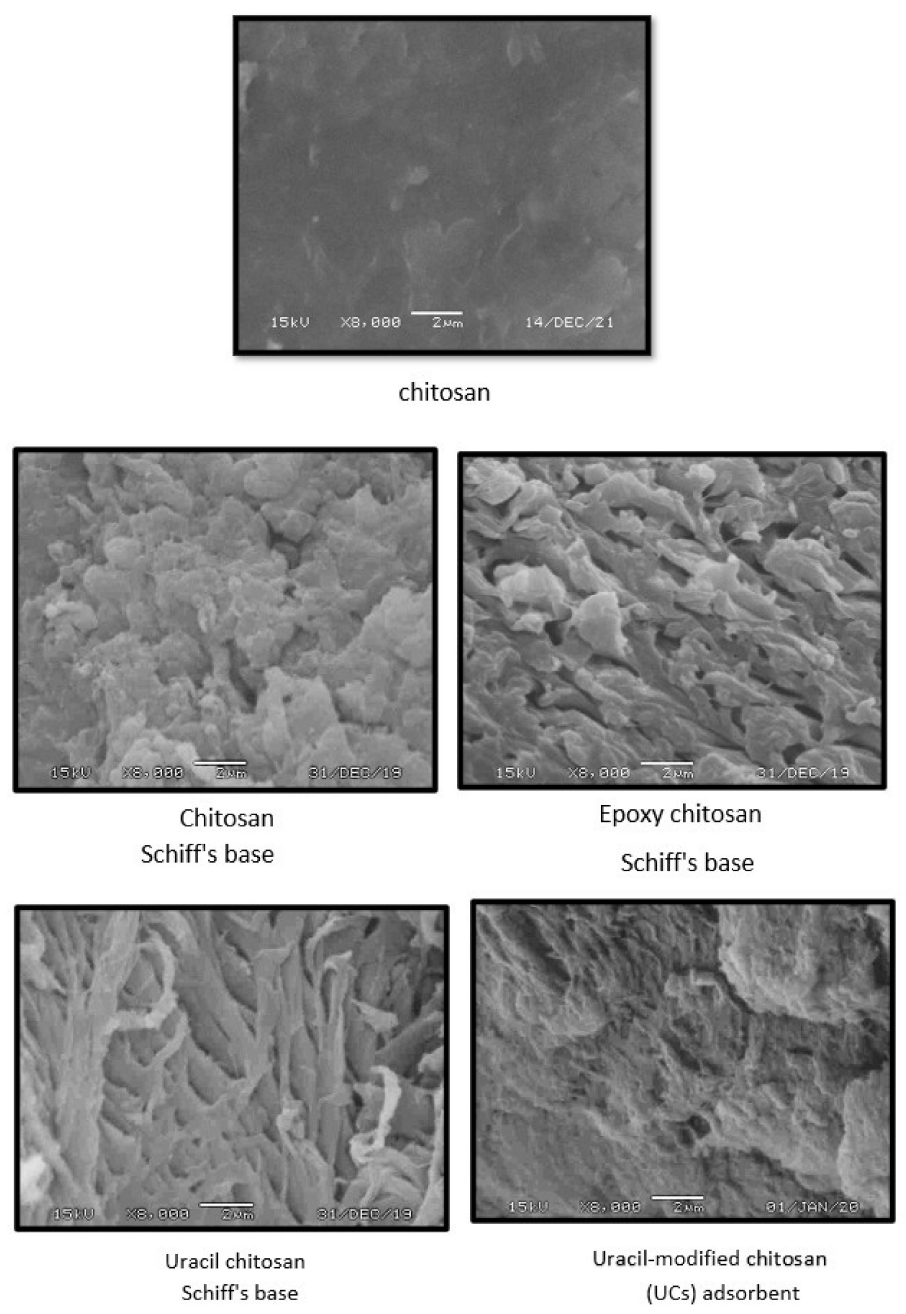
SEM images of the modified chitosan derivatives.

SEM micrographs of surface topography of the modified chitosan derivatives were shown in Figure 4. The original chitosan showed a smooth surface however its derivative showed a raucous surface, containing lumps of diverse sizes because of the incorporated substituent groups having assorted sizes. It can be noted that the lumps were homogeneously distributed over each derivative, suggesting that every stage for the chitosan modification process was successfully completed. The inserted substituent groups separate the polymer molecules away from each other, reduce the formation of their hydrogen bonds and create a porous matrix having a large surface area.

### 3.3. Adsorption of CR Dye Using UCs Adsorbent

UCs is used in the present work for the removal of CR dye from water. Its adsorption capacity comes from the incorporation of functional chelating uracil moieties, which possess more binding centers for CR dye. Throughout the study, various adsorption factors are studied, in addition to studying kinetics, isotherm and thermodynamic for understanding of the adsorption mechanism.

#### 3.3.1. Optimization of the Adsorption Conditions

##### Effect of pH

**Figure 5:**
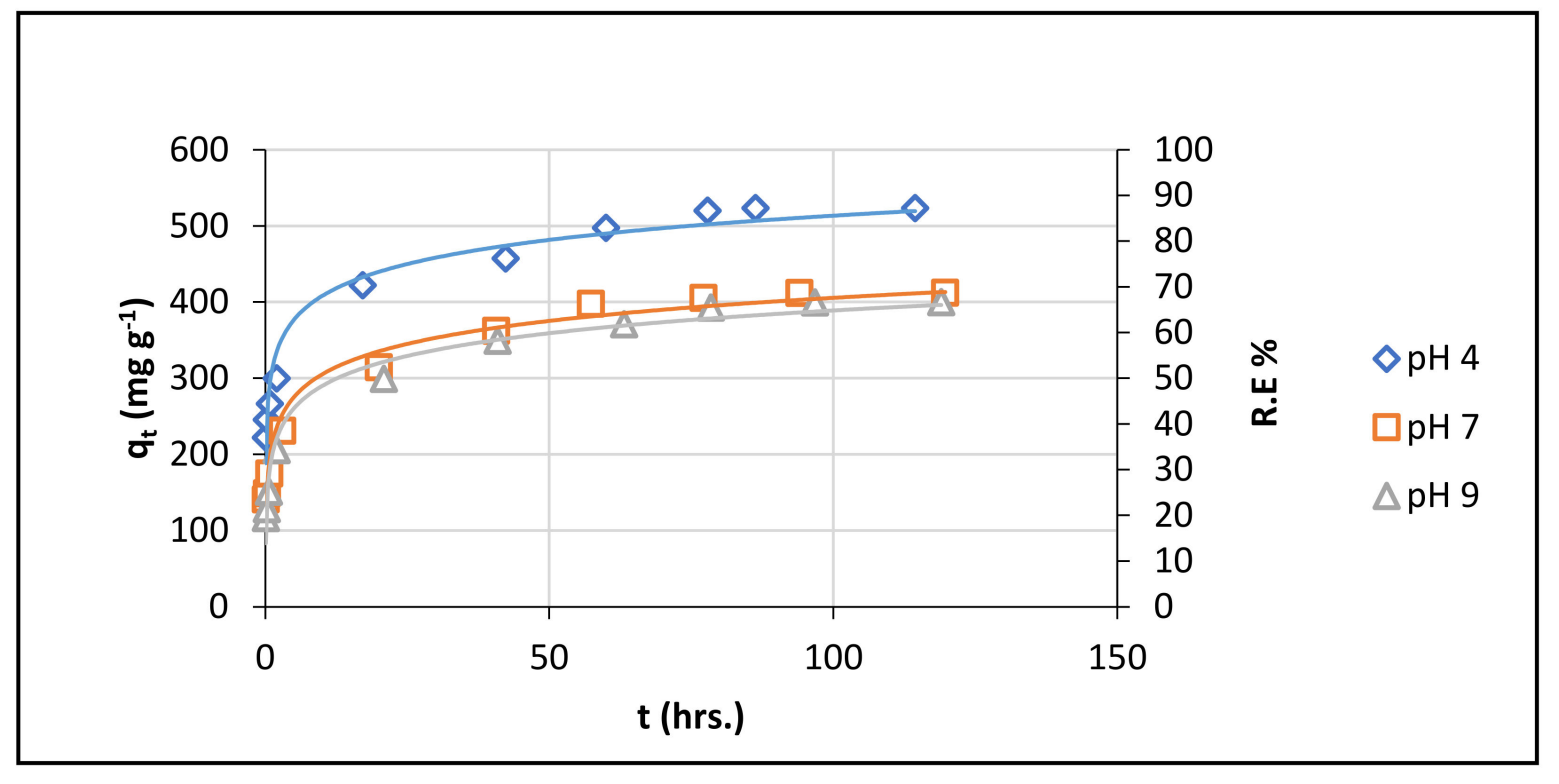
Effect of pH on CR dye adsorption onto UCs (Experimental conditions: 50 mg of adsorbent, 50 mL of dye solution (600 mg L^−1^) and temperature 55 °C).

The effect of pH on the adsorption of CR dye onto UCs adsorbent was studied at different pH values 4, 7 and 9, and the results are presented in Figure 5. The general trends for the adsorption of CR dye onto UCs reflects a continuous decreasing with increasing pH values. From the results, the percentage of CR dye removal are 87.22, 68.66 and 66.62% at pH 4, 7 and 9, respectively. This is in agreement with the study for the removal of CR dye using chitosan-coated quartz sand [25].

At low pH, an electrostatic attraction between the positively charged active sites on UCs adsorbent and dye anions takes place which leads to an increase in adsorption behavior. The incorporation of uracil moieties improves the adsorption performance of UCs, because they introduce more positive charges onto UCs surface [26].

At high pH, a strong competition between OH^−^ groups and dye anions for the positively charged sites of UCs occurs. OH^-^ groups have small size than the dye anions. Thus, binding of OH^−^ groups with UCs predominates, decreasing the adsorption performance of UCs.

Interestingly, the uracil moieties in UCs have an important role in enhancing and improving its adsorptive capacity. The % R.E. values of CR dye (adsorbent dose = 50 mg, dye solution (50 mL, 600 mg L^−1^) pH 9 and temperature 55 °C) by chitosan, chitosan Schiff’s base, epoxy chitosan schiff’s base and uracil chitosan schiff’s base were 29.7%, 20.66, 23.70 and 33.33 %, respectively, which are lower than that of UCs (66.62%).

##### Effect of Temperature

**Figure 6:**
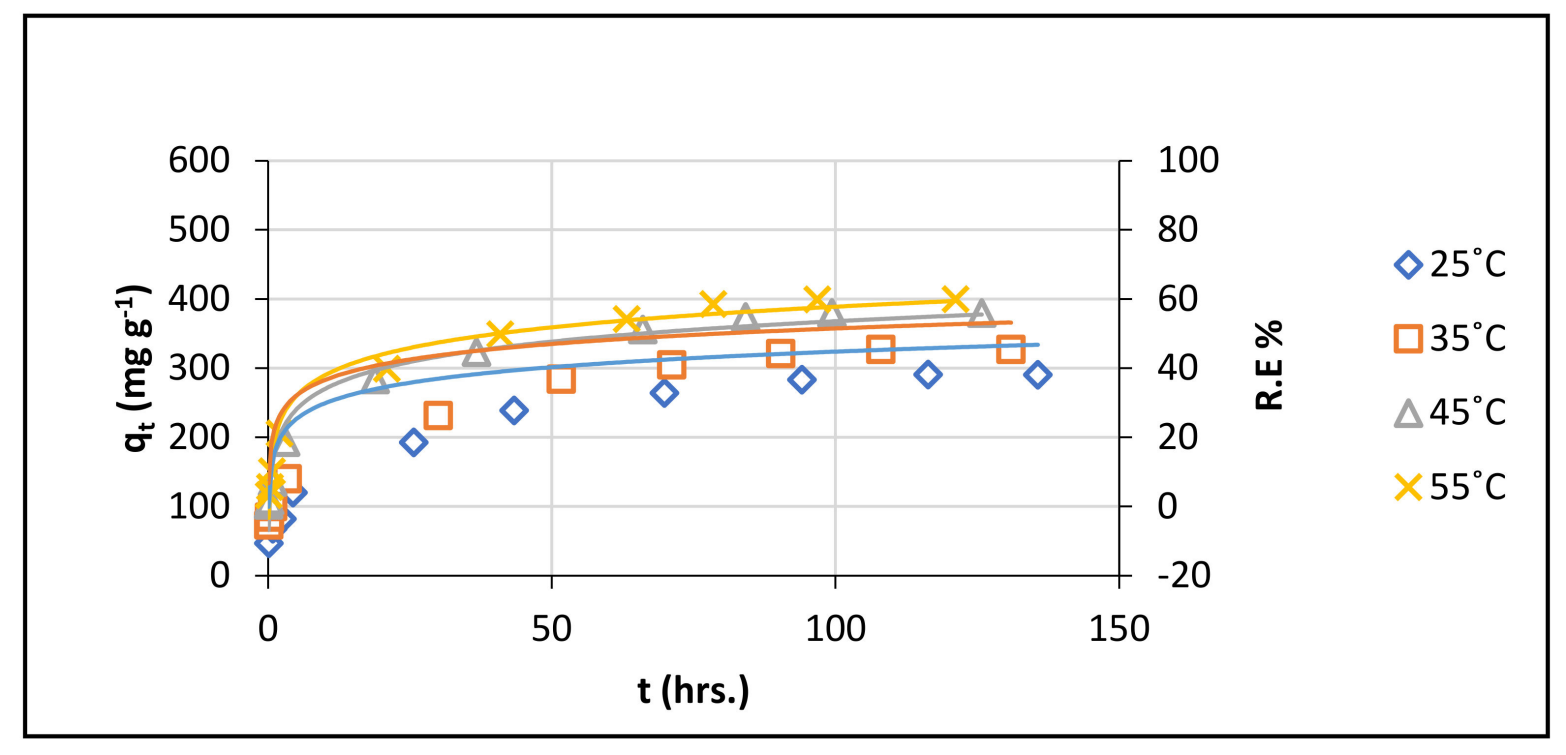
Impact of temperature on adsorption of CR dye onto UCs (Experimental conditions: 50 mg of adsorbent, 50 mL of dye solution (600 mg L^−1^) and pH 9).

Studying the effect of temperature on the adsorption of CR dye onto UCs was performed at four different temperatures (25, 35, 45 and 55 °C) and the results are illustrated in Figure 6. It is obvious that increasing the temperature was accompanied by increasing the adsorption capacity of the adsorbent towards CR dye molecules. The % R.E. values are 48.44, 54.51, 63.29 and 66.62% at temperatures 25, 35, 45 and 55 °C, respectively. This indicates that high temperature can facilitate the adsorption of CR dye onto UCs. This might be attributed to the swelling effect of the internal surface of adsorbent, which contributed for the penetration of dye molecules into the interlayer of adsorbent. This indicated that the adsorption of CR dye onto UCs was endothermic in nature [27]. Additionally, the gradual increase of temperature was followed by an increase in dye diffusivity and an increase in the dimensions of adsorbent pores. This consequently leads to reduce the contribution of intraparticle resistance and decrease the effect of boundary-layer [23,25]. The maximum adsorption capacity was achieved at 55 °C which was used as the optimum temperature for batch experiments.

##### Impact of Initial Concentration of the Dye

In the present study, the use of 150 mg L^−1^ dye solution resulted in a complete adsorption which reached to 100% of CR dye removal at pH 9, temperature 55 °C and equilibrium time 6 h.

**Figure 7:**
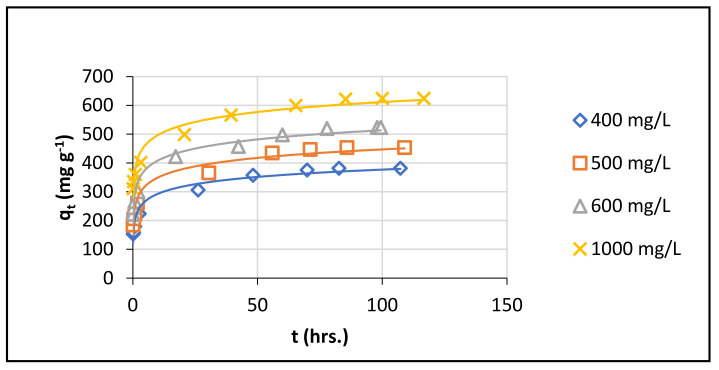

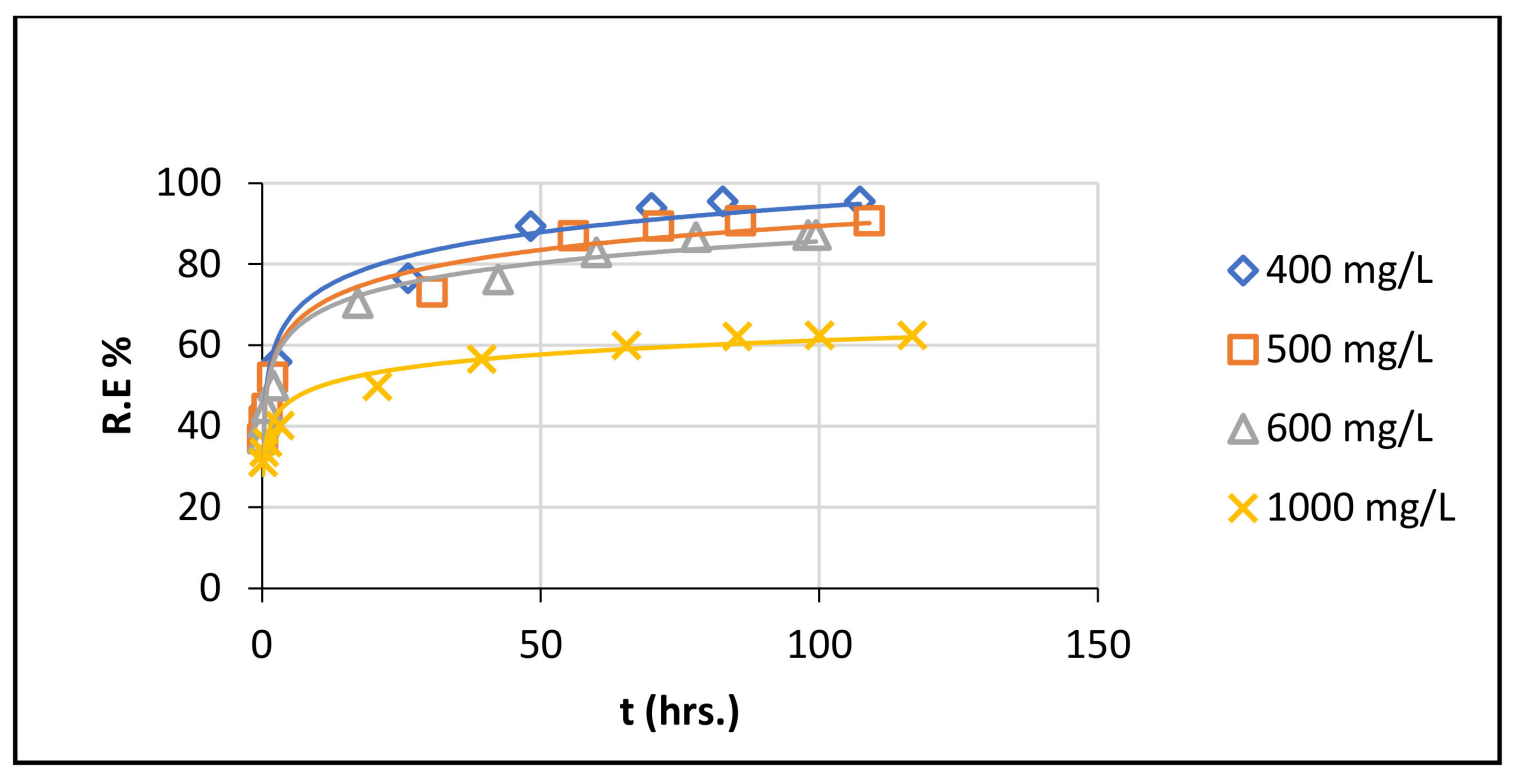
Impact of concentration of CR dye on adsorption onto UCs (Experimental conditions: 50 mg of adsorbent, 50 mL of dye solution, temperature 55 °C and pH 4).

The impact of initial concentration of the dye on its adsorption using UCs was studied at different concentrations (400, 500, 600 and 1000 mg L^−1^). The results are illustrated in Figure 7. It could be noted that increasing the initial concentration of the dye was accompanied by decreasing the removal percentage of CR dye. It decreased gradually to 95.5, 90.71, 87.22 and 62.46% at concentrations 400, 500, 600 and 1000 mg L^−1^, respectively. This can be attributed to the limited obtainable reactive centers available for adsorption process at higher concentrations. While, the adsorption capability increased with increasing initial dye concentration. This is attributed to the high driving force of the concentration gradient at higher initial CR dye concentration [25]. The maximum removal rate of CR dye was achieved at initial dye concentration of 400 mg L^−1^ which was considered as the optimum concentration.

#### 3.3.2. Adsorption Kinetics

Studying adsorption kinetics is essential for suggesting the mechanism of adsorption system. Also, it can explain the rate of dye uptake. The effect of time on the adsorption of CR dye using UCs was studied kinetically at different pH, temperatures and initial dye concentrations using three kinetic models; pseudo-first-order, Equation (5), pseudo-second-order, Equation (6), and Elovich model, Equation (7).

##### At Different pH

**Figure 8:**
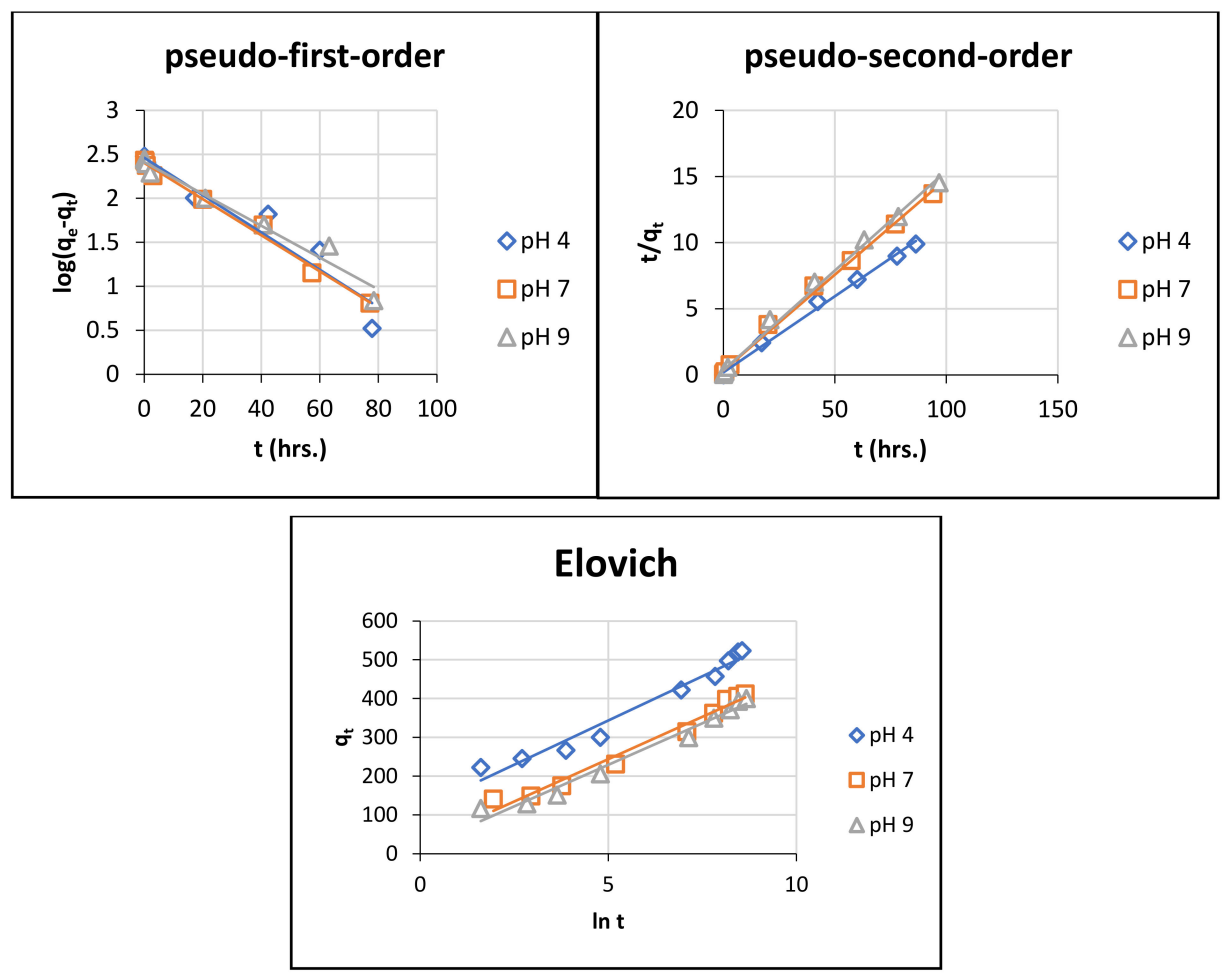
Linear regressions of kinetic plots at different pH for the adsorption of CR dye onto UCs (Experimental conditions: 50 mg of adsorbent, 50 mL of dye solution (600 mg L^−1^) and temperature 55 °C).

The results of kinetic data of the adsorption of CR dye onto UCs were summarized in Table 1. The plots of the adsorption for the three kinetic models at pH 4, 7 and 9 are illustrated in Figure 8.

The pseudo-second-order model showed the highest value of correlation coefficient (R^2^) than the other models In addition, there is a good agreement between the values of experimental and calculated q_e_ for pseudo-second-order model. This reflected and confirmed the excellent fitting of pseudo-second-order kinetic model for the adsorption process of CR dye onto UCs.

It is clearly noted that the pseudo-second-order model showed the best fit since it yielded lower values of Δ*q_e_* (%) than those obtained from pseudo-first-order at different pH (Table 1). Comparable data were published in a previous study [17].

The fitness of pseudo-second order kinetic model indicated that the adsorption process exhibited various mechanisms, including chemical interaction and electrostatic attraction of anions of the dye with the binding reactive centers onto the surface of UCs [25].

On the other hand, it is noted that the values of rate constant, k_2_, decreased gradually with increasing the pH from 4 to 9, suggesting that pH 4 was the optimum value for adsorption. At pH 4, an enhancement in the electrostatic attraction force between the protonated sites of UCs surface and the negatively charged sulfonate groups (–${\mathrm{SO}}_{3}^{-}$) of CR dye molecules takes place. This led to increase the adsorption process at low pH.

The Elovich model is one of the most interesting kinetic models that has a wide applicability to describe the adsorption systems related to the chemical nature. This model occurs when the adsorption involves the chemisorption reaction onto adsorbent surface, and the adsorption speed decreases as time passes due to covering of adsorbent surface with adsorbate [28].

Figure 8 shows the linear plots of Elovich kinetic model which obtained by applying Equation (7) at pH 4, 7 and 9. The values of α and β and the respective R^2^ are summarized in Table 1. The obtained correlation coefficients are relatively high which confirming that this model fitted fully to the results of experimental for the adsorbent. The good fitness of the experimental data for Elovich confirmed that the adsorption of CR dye onto UCs was controlled by chemisorption process.

On the other hand, it is obvious that the values of the initial adsorption rate, α, decreased with increasing pH values. As it equals 573.41–60.75 mg g^−1^ min^−1^ at pH 4 to 9. Whereas, the values of the extent of surface coverage, β, increased with increasing pH, that it ranged from 0.0219–0.0233 g mg^−1^ for the adsorption at pH 4 to 9.

##### At Different Temperatures

**Figure 9:**
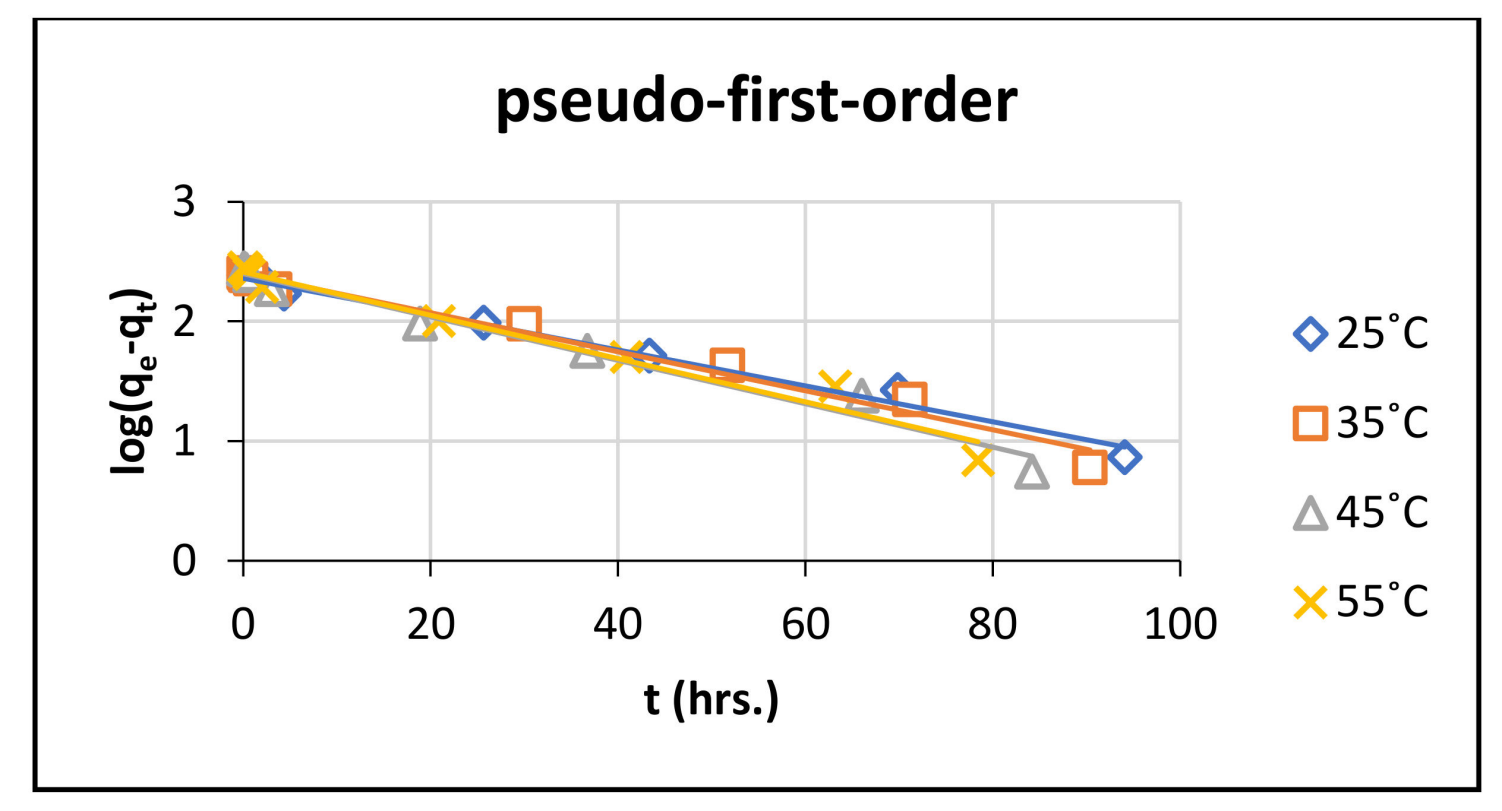

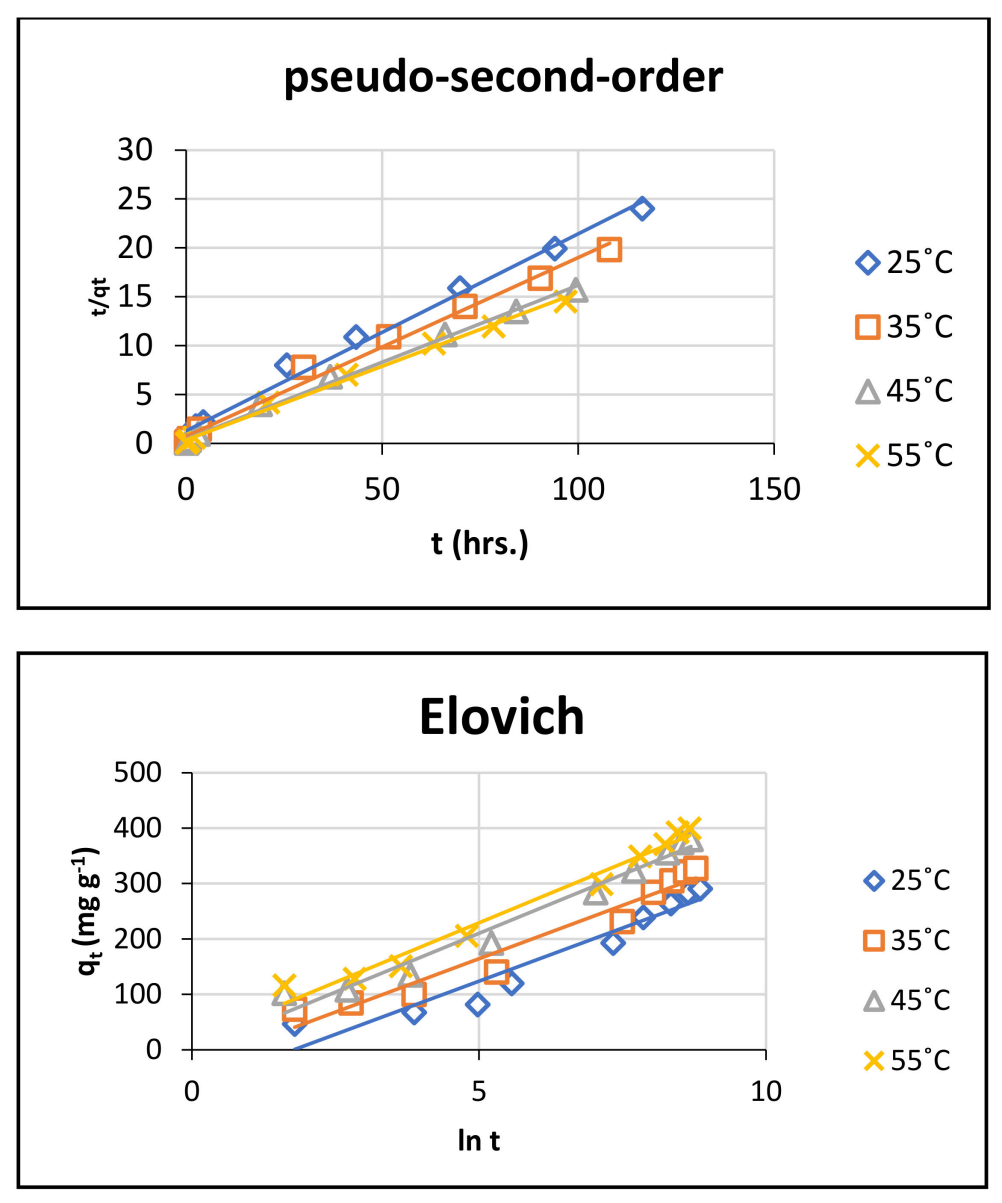
Linear regressions of kinetic plots at different temperatures for the adsorption of CR dye onto UCs (Experimental conditions: 50 mg of adsorbent, 50 mL of dye solution (600 mg L^−1^) and pH 9).

Temperature is a crucial parameter which highly affects both adsorption performance and adsorbent behavior. The experimental results of kinetic data are summarized in Table 2. The plots that showing the three kinetic models for CR dye adsorption by UCs, at 25 to 55 °C were illustrated in Figure 9.

Based on the high values of correlation coefficient (R^2^) and the low values of the normalized standard deviation Δ*q_e_* (%), it is clearly apparent that the pseudo-second-order model shows fully fit for the obtained results compared with pseudo-first-order model.

Additionally, the values of rate constant, k_2_, increased gradually by increasing the temperature, as it increased from 0.90 × 10^−5^ to 1.71 × 10^−5^ g mg^−1^ min^−1^ when temperature increased from 25 to 55 °C. This can be attributed to the low viscosity of the dye solution, which subsequently entrapped more dye molecules onto adsorbent surface [29]. Also, this might be due to the increase in collision probability between the active sites of adsorbent and the adsorbate and decreasing the boundary layer thickness of adsorbent at elevated temperature [30]. In addition, this might be due to the effect of swelling of the adsorbent internal structure, resulting from increasing temperature. This led to a more diffusion capability for dye molecules into the adsorbent surface, reaching to its internal structure, and finally increasing the rate of adsorption [27].

Figure 9 shows the linear form of Elovich model (Equation (7)). The values of the initial adsorption rate constant (α) and the desorption constant (β) at different temperatures 25, 35, 45 and 55 °C are summarized in Table 2. Obviously, the experimental data for the adsorption of CR dye onto UCs agree with Elovich model. Elovich model assumes that the adsorbent active sites were heterogenous which exhibiting various energies for chemical adsorption [28]. This finding is in agreement with the above results observed throughout the present study which concluded that pseudo-second order is the best fit model. The obtained results also suggest that the chemisorption is the rate determining step for the adsorption of CR dye onto UCs.

On the other hand, based on the results in Table 2, the values of initial adsorption rate constant, α, showed an increase with increasing temperature. Whereas, the desorption constant, β, showed a decrease with increasing temperature The decrease in the desorption constant (β) with increasing temperature is attributed to the low number of available binding sites for the adsorption at elevated temperature [31].

##### At Different Initial Dye Concentrations

**Figure 10:**
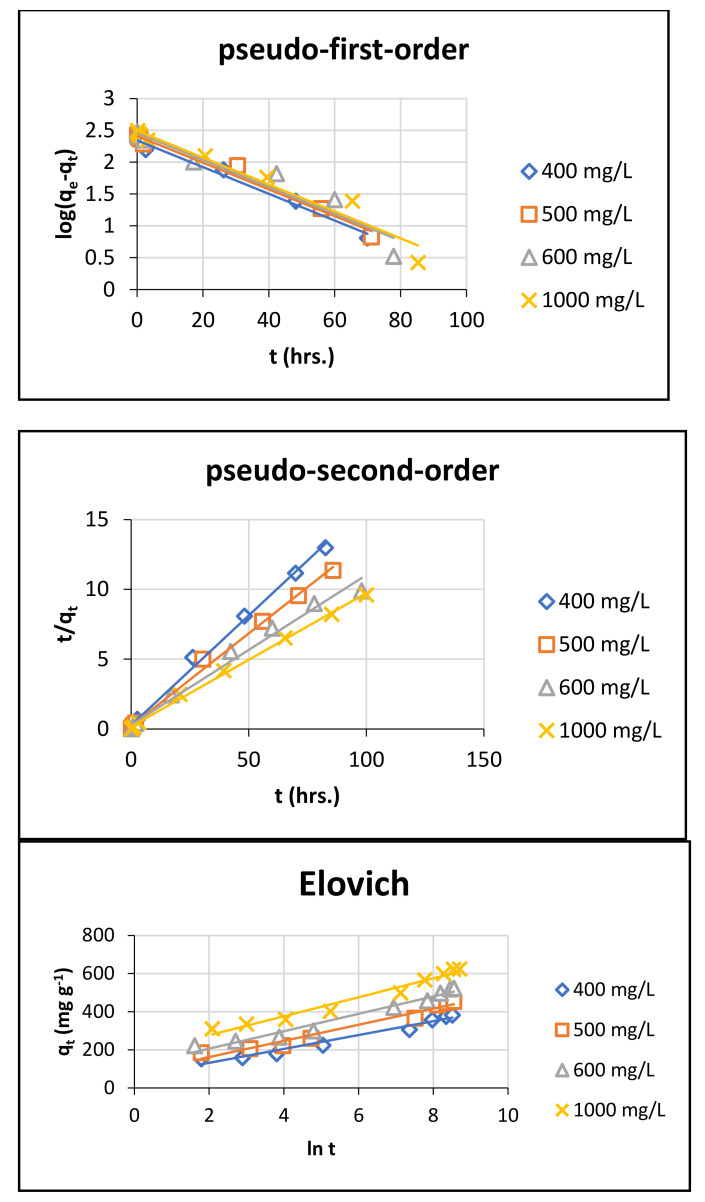
Linear regressions of kinetic plots for the adsorption of CR dye onto UCs (Experimental conditions: 50 mg of adsorbent, 50 mL of dye solution, temperature 55 °C and pH 4).

The experimental kinetic data are summarized in Table 3. Figure 10 shows the graphical linear forms of the studied three models of kinetics at different initial dye concentrations (400, 500, 600 and 1000 mg L^−1^).

It is clearly seen that the adsorption of CR dye onto the adsorbent showed excellent compliance with pseudo-second-order better than pseudo-first-order according to their respective correlation coefficients (R^2^) values and the values of the normalized standard deviation Δ*q_e_* (%). These findings indicated that the adsorption kinetics perfectly followed the pseudo-second-order model, implying that the overall rate of the adsorption process was controlled by chemisorption [30]. Similar results are obtained for adsorption of CR dye onto trimellitic anhydride isothiocyanate-cross-linked chitosan hydrogels [17].

Regarding the values of rate constants for pseudo-second-order model, k_2_, there was a significant decrease for k_2_ values with increasing dye concentrations. The same results are in agreement with previous study [31]. The obtained results confirmed that the optimum concentration value was 400 mg L^−1^. This might be ascribed to the decrease in the unoccupied available reactive centers with time, which led to decrease the adsorption rate [30].

The linear form of Elovich model, represented in Equation (7), is illustrated in Figure 10 at different dye concentrations (400–1000 mg L^−1^) for the adsorption onto UCs. The values of the initial adsorption rate α (mg g^−1^ min^−1^) and the extent of surface coverage β (g mg^−1^) are summarized in Table 3. It is noted that the experimental results agreed adequately with Elovich model. The good applicability of Elovich model indicates that the process was governed by chemisorption. On the other hand, the values of initial rate of adsorption increased with increasing concentrations. This result might be ascribed to the higher concentration gradient. Whereas, a decrease in the extent of coverage (β) with increasing concentration was observed. Since at higher concentration gradient forces the particles of CR dye to show more adsorption towards the surface of adsorbent and this might negatively affect the desorption process [30,32].

#### 3.3.3. Mechanism of CR Dye Adsorption onto UCs

Mainly, in the aqueous solution, the CR dye (CR–SO_3_Na) would dissolve and dissociate giving the dye anions (CR–SO_3_^−^) as shown in Equation (21).

(21) $$\mathrm{CR}–{\mathrm{SO}}_{3}\mathrm{Na}\;\mathrm{CR}–{\mathrm{SO}}_{3}^{-}+{\mathrm{Na}}^{+}\;$$

UCs is characterized by polar functional groups (–NH_2_ and –OH) on its surface. At low pH, the protonation of these groups takes place leading to formation of NH_3_^+^ and –OH_2_^+^, respectively. This proposes that an electrostatic interaction takes place between these two positively charged functional groups with the negatively charged dye anions as shown in Equations (22) and (23).

(22) $$–\mathrm{OH}\;\overset{H^{+}}{\to }\;–{\mathrm{OH}}_{2}^{+}\overset{\mathrm{CR}–{\mathrm{SO}}_{3}^{-}}{\to }\mathrm{CR}–{\mathrm{SO}}_{3}^{-}{\mathrm{OH}}_{2}^{+}–$$

(23) $$–{\mathrm{NH}}_{2}\overset{H^{+}}{\to }\;–{\mathrm{NH}}_{3}^{+}\overset{\mathrm{CR}–{\mathrm{SO}}_{3}^{-}\;}{\to }\;\mathrm{CR}–{\mathrm{SO}}_{3}^{-}{\mathrm{NH}}_{3}^{+}–$$

Additionally, the surface of UCs contains heterocyclic two nitrogen atoms and two carbonyl groups. Therefore, binding of CR on UCs surface via hydrogen bonding and Van der Waals forces cannot be ruled out. This proposes that the possible mechanism involves a combination of adsorbent-sorbate electrostatic interactions, between the positively charged protonated groups and negatively charged CR dye ions, in addition to H-bonding interaction and other physical forces such as π–π stacking and van der Waals forces [33]. Whereas, the electrostatic interaction might be the main mechanism for the removal behavior.

To identify and explore the diffusion mechanism for the adsorption of CR dye, the intraparticle diffusion model was applied. By studying the intraparticle diffusion, the adsorption process involves multi-step process: (i) bulk diffusion of dye molecules, (ii) film diffusion: dye molecules can diffuse to the surface of adsorbent through the boundary layer, (iii) intraparticle diffusion: dye molecules are transported from the surface into adsorbent pores and (iv) dyes are adsorbed onto the adsorbent active sites through chemical or physical reaction. Due to the continuous stirring of the batch system, ignoring diffusion by bulk could be suggested and the step that determines the rate would be the intraparticle diffusion in biosorption [34].

##### The Intraparticle Diffusion

The intraparticle diffusion model was studied for the determination of rate determining step. When the plot of qt versus t^1/2^ (Equation (8)) gives a straight-line passing via the origin; this implies that the process of adsorption is governed by diffusion, and the particle diffusion would be the rate controlling step. However, if the plot showed two or more linear regions, this indicates that the adsorption process takes place by a multistage adsorption [31].

**Figure 11:**
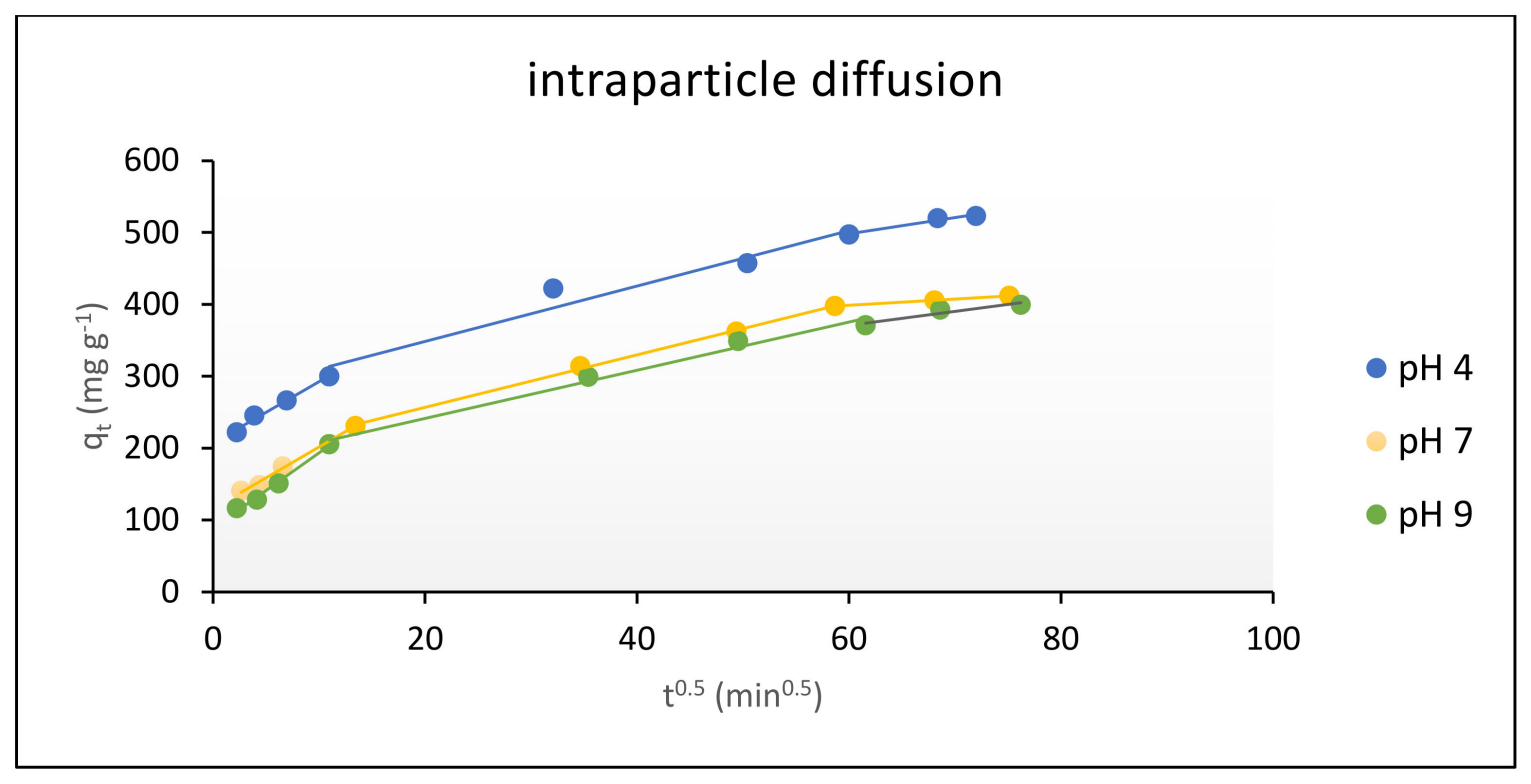
The intraparticle diffusion plots for adsorption of CR dye onto UCs using different pH values.

**Figure 12:**
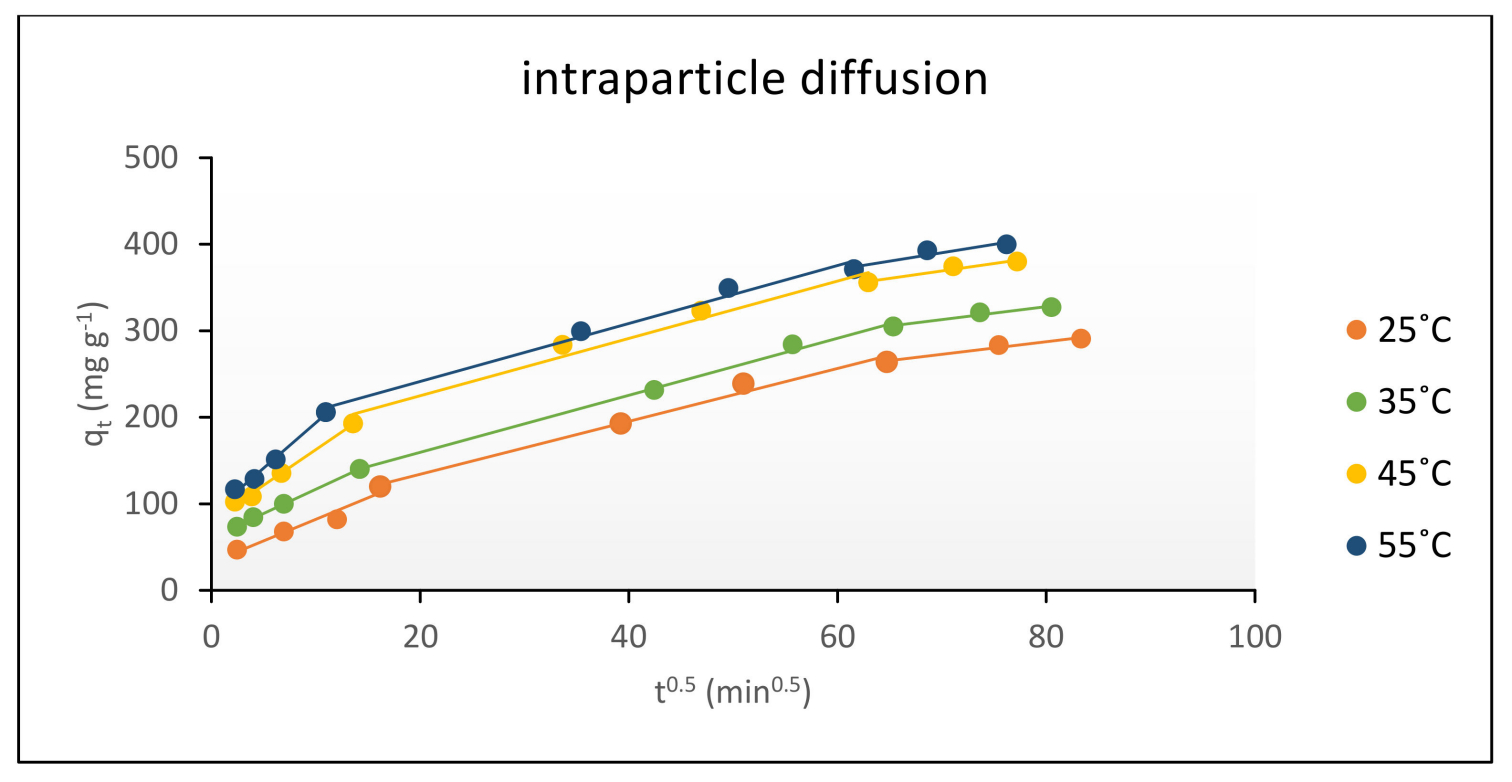
The intraparticle diffusion plots for adsorption of CR dye onto UCs at different temperatures.

**Figure 13:**
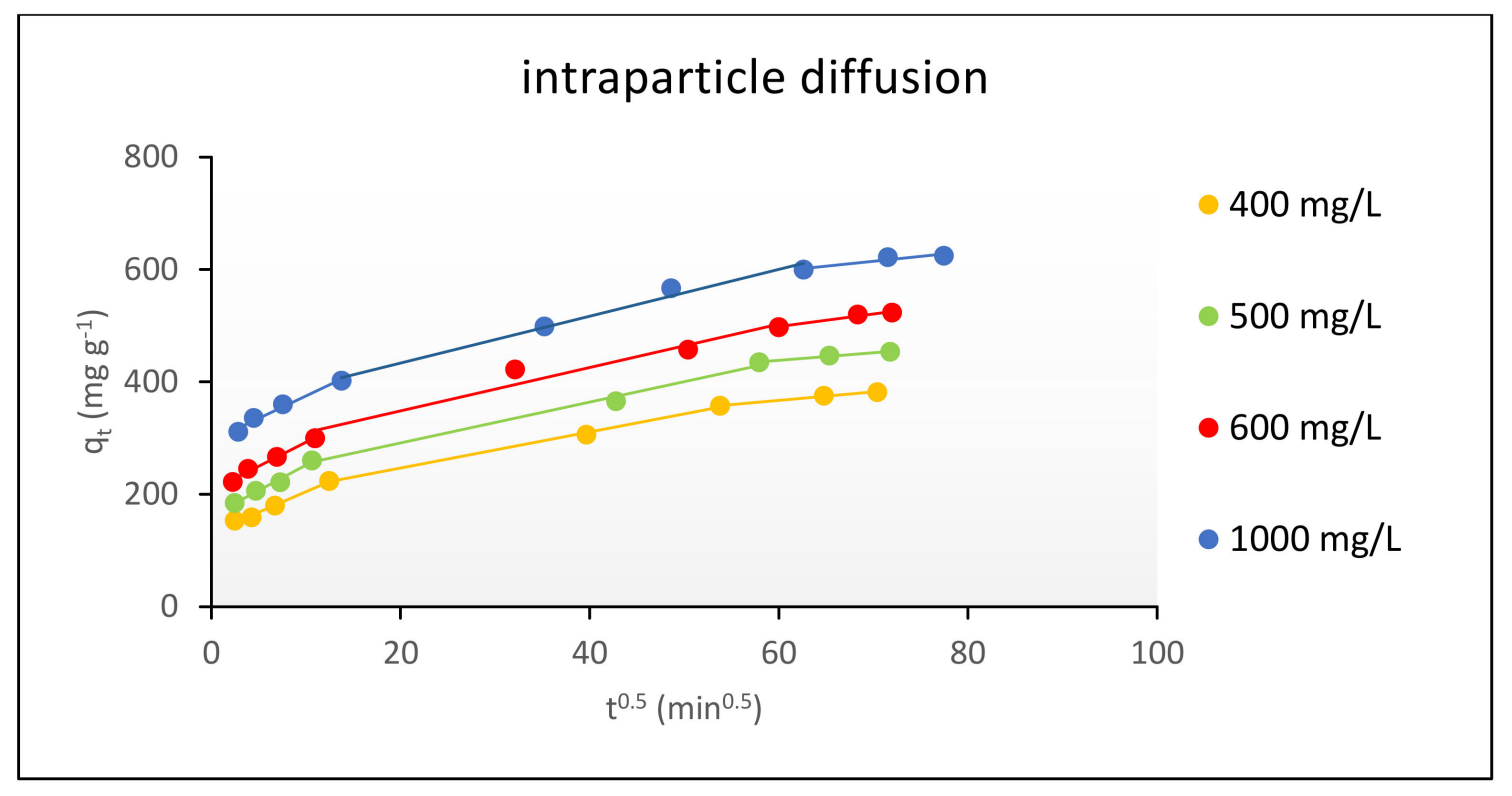
The intraparticle diffusion plots for adsorption of CR dye onto UCs using different initial concentrations.

The intraparticle diffusion results for the adsorption of CR dye onto UCs were analyzed at different pH (4 to 9), temperature (25 to 55 °C) and initial dye concentrations (400 to 1000 mg L^−^^1^), and illustrated in Figure 11, Figure 12 and Figure 13, respectively. The obtained plots didn’t pass through the origin. This confirmed that the intraparticle diffusion model was not the only rate-limiting step, suggesting other kinetic factors controlling and contributing the rate and the mechanism of adsorption. The multilinearity of the curves confirmed that the adsorption of CR dye molecules into the adsorbent surface takes place in three stages. Firstly, the dye molecule diffuses into the external surface of adsorbent by film diffusion, then in a slower step it diffuses into the internal pores of adsorbent throughout the intraparticle diffusion. The second stage was slower and more stable than the first stage, indicating that it is the step for determining the rate and the final step corresponding to the stage for final equilibrium [35].

The first slope (k_int.1_) represents the fast adsorption of dye molecules onto adsorbent surface by electrostatic interactions, while the second slope (k_int.2_) reveals to the diffusion of dye molecules into the internal pores of adsorbent. The third slope (k_int.3_) corresponded to the saturation of all active sites on adsorbent surface (equilibrium stage). These findings suggest that CR dye adsorbed onto the surface of adsorbent and in its internal structure [33].

Studying the intraparticle diffusion with the variation of pH was carried out using pH values 4, 7 and 9. The values of intraparticle diffusion rate (k_int_) and correlation coefficients (R^2^) are summarized at Table 4. It is noted that the values of k_int.2_, decreased with increasing pH values. A similar result was illustrated in a previous study for adsorption of CR dye onto pine bark [36].

The intraparticle diffusion at 25, 35, 45 and 55 °C was studied, k_int_ and R^2^ values were summarized in Table 5. The results show an increase in the diffusion rate constant with increasing temperature. The diffusion rate constant (k_int.2_) ranged from 3.06 to 3.35 mg g^−1^ min^−1/2^ when temperature increased from 25 to 55 °C, respectively. All values of R^2^ are higher than 0.9, these considerable high values suggest that the adsorption process takes place by a combination mechanism.

The intraparticle diffusion was studied also at various initial dye concentrations (400, 500, 600 and 1000 mg L^−1^) and the values of intraparticle diffusion rate (k_int_) and correlation coefficients (R^2^) are summarized in Table 6. It is found that the values of k_int_ increased with increasing the concentration. Since the values of k_int.2_ for the second stage equal to 3.21 and 4.17 mg g^−1^ min^−1/2^ at concentrations 400 and 1000 mg L^−1^, respectively. These findings indicated that the diffusion of CR dye molecules into the interior pores of adsorbent surface increases with increasing the initial dye concentration, and it is in agreement with some previous study [35].

#### 3.3.4. Adsorption Isotherm

The adsorption isotherm is considered one of the main important fundamentals for describing the mechanism of dye adsorption onto adsorbent surface. These isotherms efficiently show the way of interaction between the dye and the sorbent surface.

**Figure 14:**
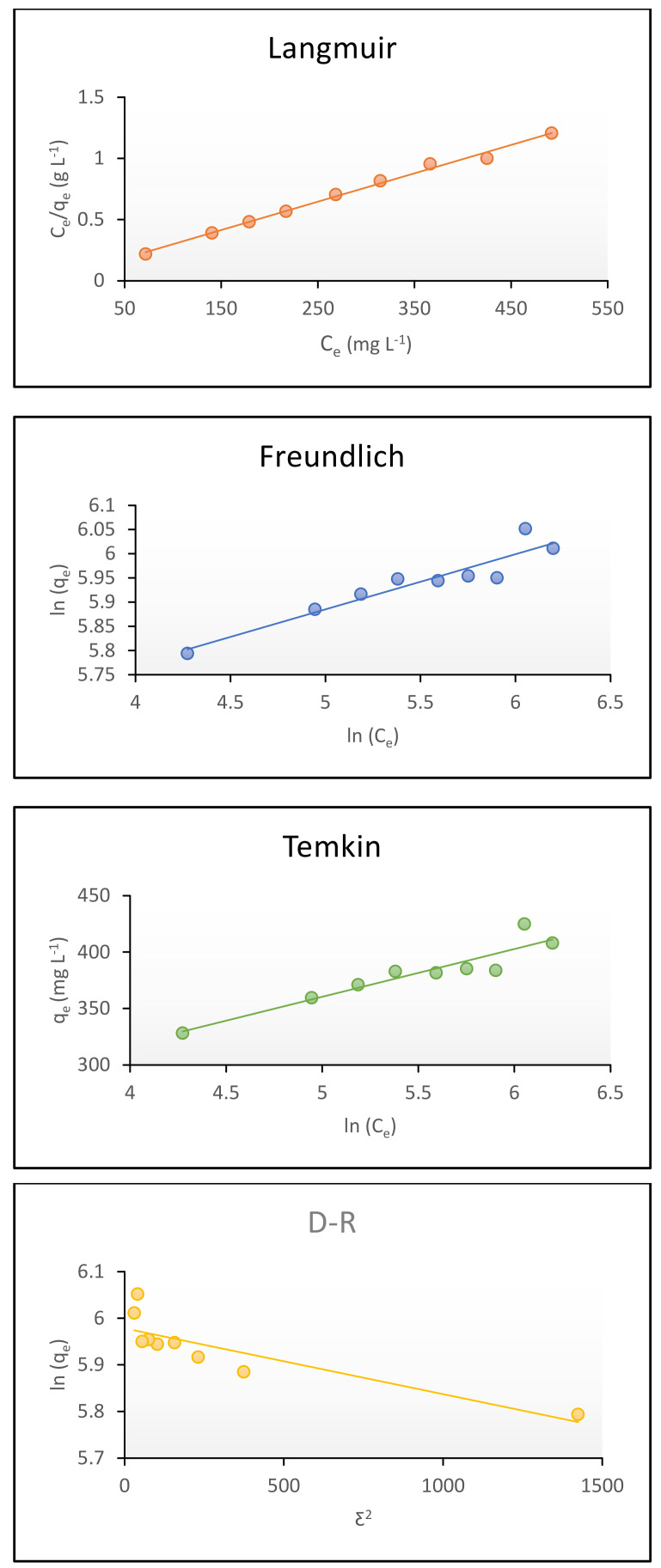
Adsorption isotherms for CR dye onto UCs at 55 °C and pH 9.

The adsorption isotherms for CR dye removal by UCs are represented in Figure 14 for Langmuir, Freundlich, Temkin and Dubinin–Radushkevich (D–R) isotherm models. Additionally, Table 7 lists the result of the four models and their corresponding fitting correlation coefficients (R^2^). The suitability of the model would be determined by the value of R^2^ which is the closest to unity [31].

According to R^2^ values, the adsorptive systems were better modeled by Langmuir adsorption isotherm than Freundlich adsorption isotherm, over the concentrations range studied. Since the value of R^2^ for Langmuir was 0.993and for Freundlich was 0.891.

The good applicability of Langmuir adsorption isotherm indicated that the adsorption process occurred in monolayer coverage manner with no interaction between adsorbed dye molecules with each other. In addition, there is a homogenous distribution of adsorption active sites onto adsorbent surface and the adsorption active sites were identical for all dye molecules. Since each active site binds only with one dye molecule [34]. The maximum monolayer coverage capacity, q_max_, at 55 °C was 434.78 mg g^−1^. Previous study also reported Langmuir model as the best fit for adsorption of dyes using chitosan [37]. The obtained R_L_ values from 0.070 to 0.032, which indicates that the adsorption of CR dye onto UCs was favorable process.

The Temkin model took into consideration the effect of indirect interaction among the molecules of adsorbate. Assuming the linear decrease of adsorption heat of all the molecules in the layer due to the interactions between adsorbent and adsorbate. The adsorption process is characterized with a uniformly distribution of binding energies [35,38].

Temkin constants, B_T_ and K_T_, are listed in Table 7. The obtained plot provided a poor curve which indicating that the adsorption process did not follow this model.

The Dubinin-Radushkevich (D-R) model is an isotherm that is used to describe the nature of adsorption process. It is similar to Langmuir, while it is more general because it rejects the homogenous surfaces [35]. The linear form of D-R isotherm (Equation (14)) is obtained by plotting lnq_e_ versus ε^2^ as illustrated in Figure 14. The values of β and ln X_m_ are calculated from the slope and intercept, respectively, and are listed in Table 7. Based on the value of R^2^, it is noted that the D-R isotherm can’t be used to describe the adsorption of CR dye onto UCs, as it equals 0.732.

#### 3.3.5. Adsorption Thermodynamics

For studying adsorption thermodynamics, the influence of temperature on the adsorption process of CR dye onto the adsorbent was examined using various temperatures (298, 308, 318 and 328 K). The values of thermodynamic parameters *ΔG°* were calculated according to Equation (17), while *ΔH°* and *ΔS°* were calculated from the Van’t Hoff linear plot (Equation (18)).

**Figure 15:**
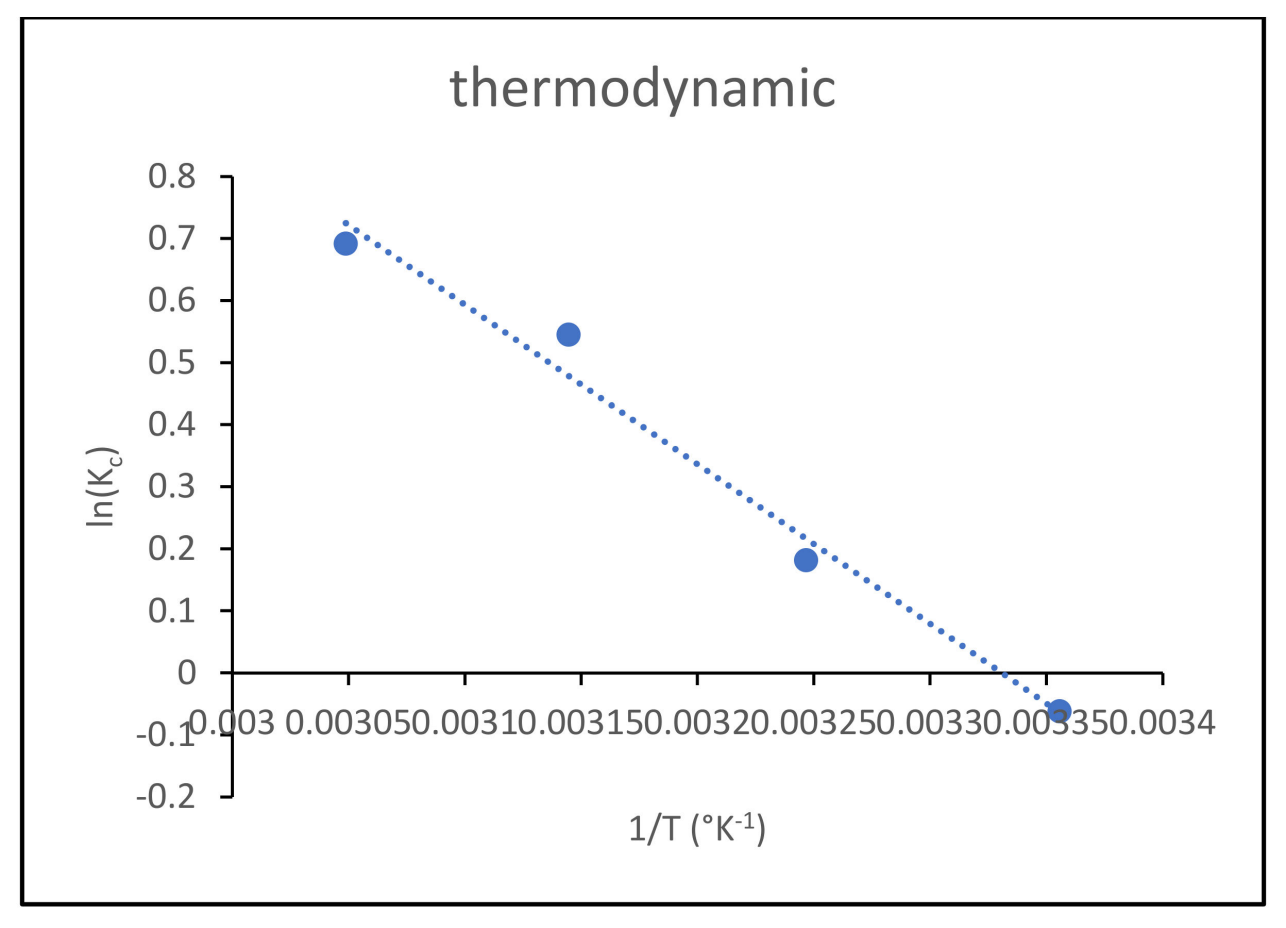
Adsorption thermodynamic of CR dye onto UCs.

The results of adsorption of CR dye onto UCs were illustrated in Figure 15 and Table 8. The values of *ΔH°* was 21.37 kJ mol^−1^, this positive value suggested that the interaction of CR dye adsorbed onto UCs was endothermic in nature [17].

The positive value of *ΔG°* at low temperature (298 K) indicating that the process was nonspontaneous and unfavorable at lower temperatures. However, at higher temperatures it showed negative values which assigned the spontaneous and favorable nature of the process [17].

The positive value of *ΔS°* (71.17 J mol^−1^K^−1^) indicated that the randomness increases at the interface between adsorbent and adsorbate solution during CR dye adsorption by UCs [37]. It can be explained as follows: throughout the adsorption process, the dye molecule receives more entropy due to the displacement of adsorbed solvent molecule which resulting finally with increasing randomness [39]. This actually occurred due to increasing the number of free ions of adsorbate that were found in order form near adsorbent surface than the adsorbed ions before adsorption process. Hence, the distribution of rotational energy increased, that consequently increases randomness at the interface between solid and liquid, and finally results in increasing adsorption [30]. These findings were in a good agreement with previous study [37].

#### 3.3.6. Activation Energy

**Figure 16:**
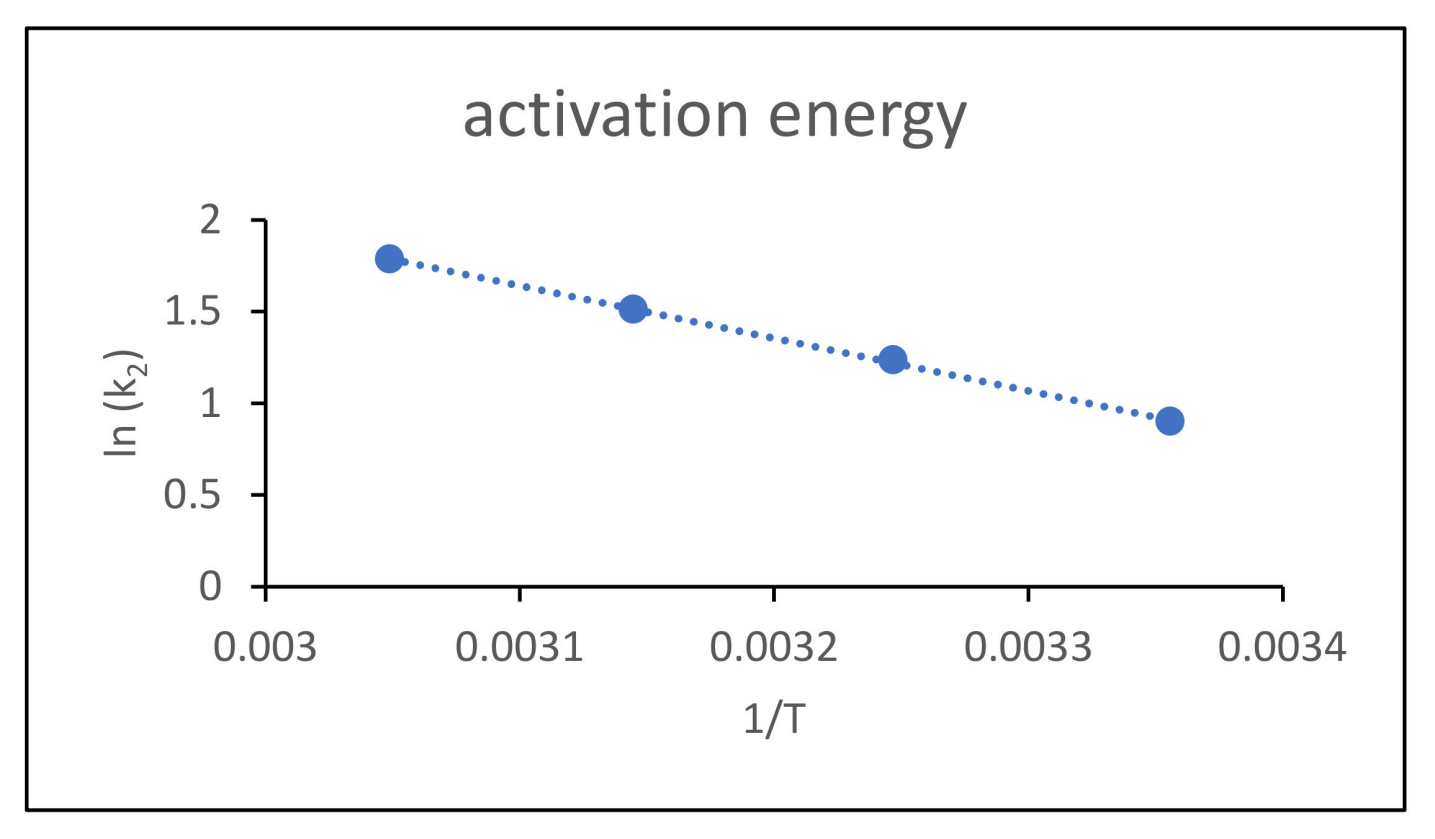
Arrhenius plot for adsorption of CR dye onto UCs.

The type of adsorption process whether it is physisorption or chemisorption can be determined by the magnitude of activation energy. When the activation energy is found in the range 0–40 kJ mol^−1^, this indicates that the reaction is physisorption, while if it is in the range 40–800 kJ mol^−1^, the reaction is chemisorption [18]. The value of E_a_ for the adsorption of CR dye onto UCs was 18.40 kJ mol^−1^, as illustrated in Figure 16. This value indicates that the adsorption was a physisorption in nature and was ascribed to a low potential barrier [40]. This result agrees with the adsorption of CR dye onto activated Moringa oleifera seed [41] and onto guava leaf-based activated carbon [31].

#### 3.3.7. Comparison between UCs and Other Adsorbents for CR Dye Removal

To study the affinity and efficiency of the UCs adsorbent, it became of interest to compare its maximum capacity of adsorption for removal of CR dye with other adsorbents in previous reported studies as shown in Table 9. It is clearly observed that UCs possesses the highest removal capacities for CR dye than other reported adsorbents. It could efficiently adsorb CR dye at 55 °C with q_max_ of 624.67 mg g^−1^. This proves that UCs is efficient and promising adsorbent for the removal of CR dye from aqueous solution.

#### 3.3.8. Desorption Studies

The reusability is considered one of the most important aspects to minimize the cost of adsorption by regeneration of adsorbent [47,48].

The desorption of CR dye was applied using Equation (20). No desorption was obtained by using ethanol, methanol and acetone as desorption medium, while using NaOH showed that the desorption percentage reached to 24% after 5 cycles. These findings confirmed that UCs can be efficiently reused for the adsorption of CR dye from aqueous solution.

## 4. Conclusions

Chitosan has been modified by a procedure comprising four steps involving the incorporation of uracil moieties at the last step. The obtained uracil-modified chitosan (UCs) contained free primary amino and hydroxyl active centers in its main chains, in addition to nitrogen- and oxygen rich uracil nuclei as pendant groups on its repeating units. Its structure was characterized by different techniques; FTIR, XRD and SEM. Its active basic groups act as efficiently binding sites for the anionic CR dye from its aqueous solution. The adsorption capacity for CR dye onto UCs increased with increasing temperature reaching its maximum at 55 °C. The adsorption capacity decreased with increasing pH achieving its optimum value at pH 4. The removal percentage of CR dye decreased with increasing the initial concentration of the dye. The pseudo-second-order model showed a perfect fit to describe the adsorption process, indicating the chemisorption process. The values of rate constant, k_2_, decreased gradually with increasing the pH from 4 to 9 and initial concentration of dye solution from 400 to 1000 mg L^−1^, while it increased with increasing temperature from 25 °C to 55 °C. The relatively high correlation coefficient R^2^ obtained from Elovich model confirmed that this model fitted fully to the experimental results for the adsorbent at the studied parameters. This Indicates that the adsorption of CR dye onto UCs was controlled by chemisorption process. According to intraparticle diffusion, the transfer of CR dye molecules into the adsorbent surface takes place in three stages. The plots of intraparticle diffusion showed that it was not the only rate-limiting step. The good applicability of Langmuir adsorption isotherm indicated that the adsorption process takes place in monolayer coverage manner with no interaction between adsorbed dye molecules with each other. The maximum monolayer coverage capacity, q_max_, at 55 °C was 434.78 mg g^−1^. Freudlich and Temkin models didn’t show high accuracy as their correlation coefficients weren’t as high as Langmuir isotherm. Whereas, the experimental data confirms the unsuitability of the Dubinin–Radushkevich isotherm model. The positive value of *ΔH°* (21.37 kJ mol^−1^) suggests that the adsorption was endothermic in nature. The positive value of *ΔG°* at low temperature indicating that the process was nonspontaneous at lower temperatures. However, at higher temperatures it showed negative values which assigned the spontaneous process. The positive value of *ΔS°* indicates the randomness elevation at the interface between adsorbent and adsorbate solution. The value of activation energy *E_a_* was 18.40 kJ mol^−1^, assigning that the adsorption was physisorption. Thus, it is concluded that the adsorption of CR dye UCs involved physisorption and chemisorption processes.

## Figures and Tables

**Table 1:** Kinetic model constants and correlation coefficients for the adsorption of CR dye onto UCs at different pH.

Kinetic Models	Parameters	PH		
		4	7	9
q_e.exp_ (mg g^−1^)		523.33	412	399.78
pseudo- first-order	R^2^	0.934	0.988	0.969
	q_e.cal_ (mg g^−1^)	289.27	253.16	258.82
	K_1_ (10^−4^) (min^−1^)	9.21	6.91	6.91
	Δq_e_ (%)	15.81	13.36	12.47
pseudo- second-order	R^2^	0.996	0.996	0.995
	q_e.cal_ (mg g^−1^)	526.32	416.67	400
	K_2_ (10^−5^) (g mg^−1^ min^−1^)	1.82	1.72	1.71
	Δq_e_ (%)	0.201	0.400	0.019
Elovich	R^2^	0.967	0.975	0.976
	β (g mg^−1^)	0.0219	0.0228	0.0233
	α (mg g^−1^ min^−1^)	573.41	78.52	60.75

**Table 2:** Kinetic model constants and correlation coefficients for the adsorption of CR dye onto UCs at different temperatures.

Kinetic Models	Parameters	Temperatures			
		25 °C	35 °C	45 °C	55 °C
q_e.exp_ (mg g^−1^)		290.67	327.11	379.78	399.78
pseudo-first-order	R^2^	0.987	0.979	0.975	0.969
	q_e.cal_ (mg g^−1^)	229.88	250.78	251.88	258.82
	K_1_ (10^−4^) (min^−1^)	4.61	6.91	6.91	6.91
	Δq_e_ (%)	7.39	8.25	11.91	12.47
pseudo-second-order	R^2^	0.990	0.990	0.995	0.995
	q_e.cal_ (mg g^−1^)	294.12	333.33	384.62	400.0
	K_2_ (10^−5^) (g mg^−1^ min^−1^)	0.90	1.24	1.54	1.71
	Δq_e_ (%)	0.42	0.67	0.45	0.02
Elovich	R^2^	0.922	0.951	0.970	0.976
	β (g mg^−1^)	0.026	0.0259	0.0235	0.0233
	A (mg g^−1^ min^−1^)	6.55	18.69	40.65	60.75

**Table 3:** Kinetic model constants and correlation coefficients for the adsorption of CR dye onto UCs at different dye concentrations.

Kinetic Models	Parameters	Dye Concentrations			
		400 mg L^−1^	500 mg L^−1^	600 mg L^−1^	1000 mg L^−1^
q_e.exp_ (mg g^−1^)		382.0	453.56	523.33	624.67
pseudo- first-order	R^2^	0.988	0.980	0.934	0.953
	q_e.cal_ (mg g^−1^)	219.43	258.40	289.27	312.32
	K_1_ (10^−4^) (min^−1^)	6.91	6.91	9.21	9.21
	Δ*q_e_* (%)	16.09	16.26	15.81	17.68
pseudo- second-order	R^2^	0.995	0.995	0.996	0.997
	q_e.cal_ (mg g^−1^)	384.62	454.55	526.32	625.0
	K_2_ (10^−5^) (g mg^−1^ min^−1^)	2.46	2.20	1.82	1.46
	Δ*q_e_* (%)	0.26	0.08	0.20	0.019
Elovich	R^2^	0.965	0.964	0.967	0.966
	β (g mg^−1^)	0.0274	0.0235	0.0219	0.0201
	α (mg g^−1^ min^−1^)	183.53	263.83	573.41	1765.75

**Table 4:** Parameters of intraparticle diffusion model using different pH for adsorption of CR dye onto UCs.

	Parameters		PH	
		4	7	9
Whole Processes	R^2^	0.969	0.960	0.961
	k_i_	4.22	3.86	3.86
1st	K_i.1_	9.15	8.84	8.80
2nd	K_i.2_	3.85	3.65	3.35
3rd	K_i.3_	0.92	0.91	0.91

**Table 5:** Parameters of intraparticle diffusion model for adsorption of CR dye onto UCs at different temperatures.

	Parameters	Temperatures			
		25 °C	35 °C	45 °C	55 °C
Whole Processes	R^2^	0.971	0.984	0.953	0.961
	k_i_	3.12	3.35	3.76	3.86
1st	K_i.1_	3.64	5.82	7.69	8.80
2nd	K_i.2_	3.06	3.30	3.31	3.35
3rd	K_i.3_	0.87	0.87	0.90	0.91

**Table 6:** Parameters of intraparticle diffusion model for adsorption onto UCs using different CR dye concentrations.

	Parameters	Dye Concentrations (mg L^−1^)			
		400	500	600	1000
Whole processes	k_i_	3.42	3.86	4.22	4.22
1st	K_i.1_	6.40	7.68	9.15	10.04
2nd	K_i.2_	3.21	3.62	3.85	4.17
3rd	K_i.3_	1.14	1.04	0.92	0.45

**Table 7:** Adsorption isotherm constants of CR dye by UCs.

Isotherm Model	Parameters	
Langmuir	R^2^	0.993
	K_L_ (L mg^−1^)	0.033
	q_max_ (mg g^−1^)	434.78
	R_L_	0.070–0.032
Freundlich	R^2^	0.891
	K_f_ (L mg^−1^)	203.41
	1/n	0.114
Temkin	R^2^	0.876
	K_T_ (L mg^−1^)	33.54
	B (J mol^−1^)	42.34
D-R	R^2^	0.732
	X_max_ (mg g^−1^)	394.85
	β (mol^2^ J^−2^)	0.0001
	E (kJ mol^−1^)	0.07

**Table 8:** Parameters of thermodynamic of adsorbing CR dye by UCs.

Thermodynamic Parameters	Temperature (K)	
*∆G*° (KJ mol^−1^)	298	0.15
	308	−0.46
	318	−1.44
	328	−1.89
*ΔH*° (kJ mol^−1^)	21.37	
*ΔS*° (J K^−1^ mol^−1^)	71.17	

**Table 9:** Comparison of adsorption capacity of UCs with other adsorbents for the removal of CR dye.

Adsorbent	Adsorption Capacity (mg g^−1^)	Temperature °C	Dye Concentration mg L^−1^	Adsorbent Dose (g)	pH	Ref.
Tunics of the corm of the saffron	6.2	25	50–500	0.5	10	[42]
Quaternized chitosan/chitosan cationic polyelectrolyte microsphere	1500	25	0–1000	0.075	5	[43]
Activated carbon coffee waste	90.90	25	50 mg	0.1	3	[44]
xanthated chitosan/cellulose sponges	289.855	30	6.25–200	0.05	6	[45]
Chitosan and Laponite based nanocomposite	390.3	30	500	1	6	[46]
UCs	624.67	55	1000	0.05	4	Present study

## Data Availability

The data presented in this study are available on request from the corresponding author.
